# Immobilization-free SELEX for aptamer discovery targeting colorectal cancer-derived small extracellular vesicles

**DOI:** 10.1186/s12951-025-03813-0

**Published:** 2025-11-25

**Authors:** Eun Sung Lee, Byung Seok Cha, Junhyeong Kim, Seung Hyeon Reo, Jinseo Son, Ki Soo Park

**Affiliations:** 1 Department of Biological Engineering, College of Engineering, Konkuk University, Seoul, 05029 Republic of Korea; 2 Advanced Materials Program, Department of Biological Engineering, Konkuk University, Seoul, 05029 Republic of Korea

**Keywords:** Small extracellular vesicles, SELEX, Aptamer, Cancer diagnosis, Colorectal cancer, Isothermal amplification

## Abstract

**Background:**

Despite the increasing prominence of small extracellular vesicles (sEVs) and liquid biopsies for early cancer diagnosis, the development of high-performance molecular probes specifically targeting sEVs remains limited. In this study, we present a novel enzymatic digestion sEV-systematic evolution of ligands by exponential enrichment (EDGE-SELEX) strategy that eliminates the need for sEV immobilization, thereby preserving the native structural and biochemical characteristics of sEVs and better mimicking their clinical environment.

**Results:**

Using the EDGE-SELEX approach combined with a post-selection optimization process, we successfully identified two novel aptamers, H7F-3 and H15F, exhibiting high affinity for colorectal cancer (CRC)-derived sEVs, with dissociation constants of 8.149 and 3.347 nM, respectively. Structural analysis suggested that the G6 motif plays an important role in aptamer-sEV binding. This motif also demonstrated potential for incorporation into split and blocking aptamer designs. Furthermore, we developed an aptamer-based loop-mediated isothermal amplification for sEV detection (ABLE) system, which achieved detection limit of 20 particles/µL for CRC-derived sEVs.

**Conclusions:**

Our findings demonstrate the applicability of SELEX technology to native sEVs and highlight the diagnostic potential of the identified aptamers for sEV-based cancer detection. The EDGE-SELEX method and the G6 motif may serve as valuable tools for future clinical applications in non-invasive cancer diagnostics and aptamer engineering, although further validation with clinical samples is warranted.

**Graphical abstract:**

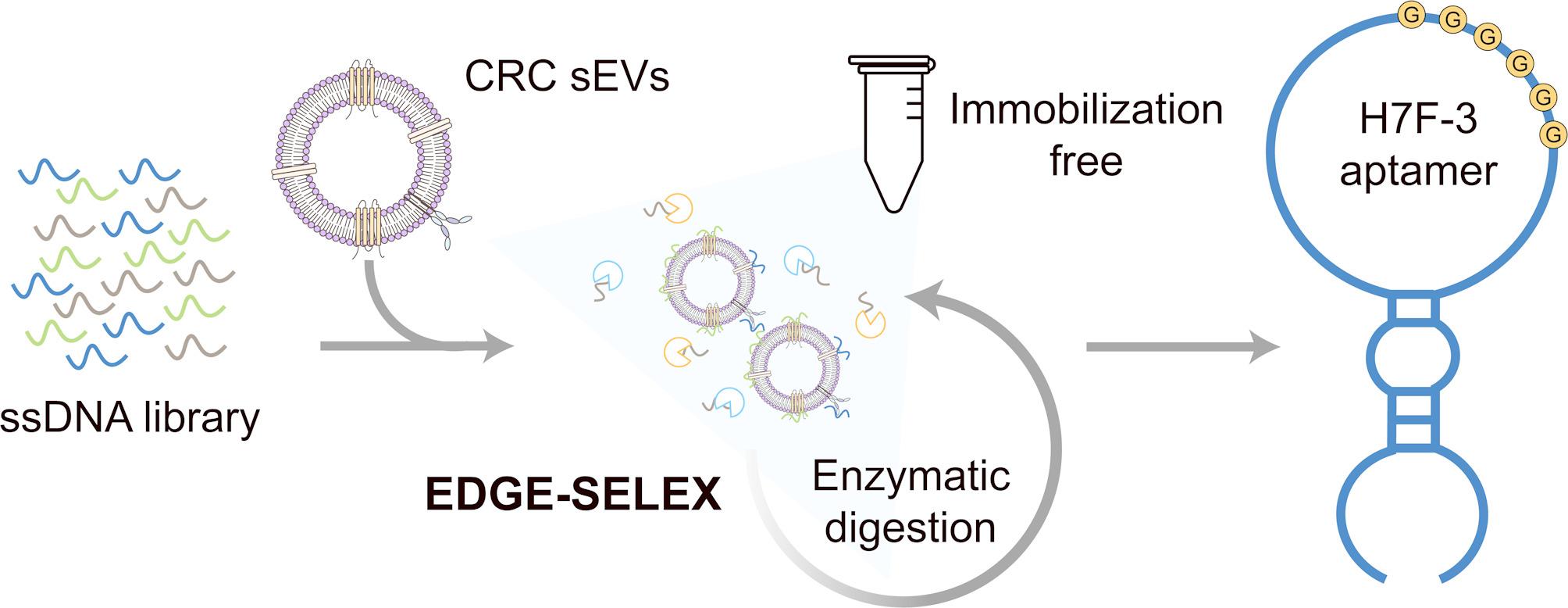

**Supplementary Information:**

The online version contains supplementary material available at 10.1186/s12951-025-03813-0.

## Background

Tissue biopsy remains the gold standard for diagnosing cancer and determining the cause of abnormalities, typically requiring the surgical removal of a small tissue sample from the body [1, 2]. However, tissue biopsy is invasive and procedurally complex. Notably, liquid biopsy has emerged as a promising alternative [1], offering a less invasive method for sample acquisition and minimizing patient burden. Theoretically, various body fluids, such as blood, saliva, or urine, can serve as sources for liquid biopsy, as they contain circulating tumor cells, circulating tumor DNA, or extracellular vesicles (EVs) [3–5]. In particular, EVs have garnered substantial attention as valuable targets for liquid biopsy, as they are released by nearly all cell types and are abundant in body fluids [6, 7]. Among the different types of EVs, small EVs (sEVs) are crucial mediators of intercellular communication [8–11]. These sEVs range from 50 to 150 nm in size and comprise phospholipid bilayer nanovesicles containing proteins and nucleic acids derived from their parent cells. Notably, sEV analysis enables the tracing of their cellular origins, facilitating early and minimally invasive cancer diagnosis [12–15]. However, a vast majority of circulating sEVs are derived from erythrocytes and epithelial cells, and tumor-derived sEVs constitute only a small fraction [16–18]. These critical issues highlight the urgent need to develop advanced technologies to accurately identify tumor-derived sEVs, with special emphasis on designing novel targeting probes.

Emerging evidence highlights the importance of sEV profiling and detection in early cancer diagnosis, which requires the development of sEV-targeting probes, such as antibodies and aptamers [19–21]. Aptamers are single-stranded DNA (ssDNA) or RNA oligonucleotides that bind specifically to target molecules [22]. They exhibit notable biocompatibility, ease of modification, cost-effectiveness, and high binding affinity. Aptamers can be developed through the systematic evolution of ligands by exponential enrichment (SELEX) process, which can be tailored to a diverse range of targets, from biomolecules, such as proteins and cells, to small molecules [23–27]. SELEX has been used for the selection of aptamers specific to sEVs [20, 28–30]. Although promising, this process requires immobilization of the target sEVs. However, immobilization does not accurately reflect physiological conditions, as the surface proteins of sEVs exhibit substantial differences in exposed epitopes and surrounding molecular environments between their isolated form and their embedded (native) state within phospholipid bilayers [31–33]. This issue is evidenced by the variable binding affinities of representative targeting ligands, such as antibodies, depending on the state of the protein samples. This is observed in techniques such as enzyme-linked immunosorbent assay, western blotting, and immunohistochemistry [34–36]. Therefore, a new SELEX process that does not require the immobilization of sEVs is essential to reflect the native state of protein markers present on sEV surfaces in clinical environments, thus ensuring superior binding affinity.

In the present study, we developed an enzymatic digestion sEV-SELEX (EDGE-SELEX) technology that avoids sEV immobilization while conserving the native environment of the proteins present on the sEV membrane surface. This approach relies on the unique features of exonuclease I (Exo I) and T5 exonuclease (T5 exo) to digest ssDNA that is not bound to sEVs. We selected the colorectal cancer (CRC) cell line, HT29, as the model system and screened DNA aptamers specific to HT29-derived sEVs (HT29 sEVs). Among the selected aptamer candidates, H7 and H15 showed high binding affinity and were further optimized through a post-SELEX process. The optimal aptamer sequences of H7F-3 and H15F showed dissociation constants in the nanomolar range with high binding specificity. Additionally, we identified a G6 motif within the H7F-3 and H15F aptamers that was important for binding to target sEVs. Finally, we established an aptamer-based loop-mediated isothermal amplification for sEV detection (ABLE) system, which exhibited high sensitivity with a detection limit of 20 particles/µL.

## Methods

### Cell culture

The cell lines used in this study were purchased from the Korean Cell Line Bank (Seoul, Republic of Korea). HT29, SW620, and CCD-18Co cells were cultured in Dulbecco’s modified Eagle’s medium (Welgene, Gyeongsan, Republic of Korea). SKBR3 cells were cultured in Roswell Park Memorial Institute medium (Welgene). HeLa and U-87 MG cells were cultured in minimum essential medium (Welgene). All media were supplemented with 10% v/v fetal bovine serum (FBS; Gibco, Thermo Fisher Scientific, Waltham, MA, USA) and 1% v/v penicillin-streptomycin (Welgene). All cell lines were incubated at 37 °C in a humidified atmosphere containing 5% CO_2_.

### sEV isolation and purification

After reaching approximately 80% confluence in 175 T flasks (Sarstedt, Nümbrecht, Germany), the media were replaced with fresh media containing 5% v/v EV-depleted FBS (Gibco) and 1% v/v penicillin-streptomycin (Welgene). The cells were incubated for 48 h in a CO_2_ incubator, and the media were collected to isolate and purify the sEVs. The collected media were subjected to a series of differential centrifugation steps to remove large molecules, such as cells, cell debris, apoptotic bodies, and microvesicles: 300 ×*g* for 5 min, 2,000 ×*g* for 20 min, and 10,000 ×*g* for 1 h. The supernatants were filtered through 0.45- and 0.22-µm pore size filters (Sartorius AG, Göttingen, Germany) to eliminate molecules larger than 200 nm in size. Further purification and concentration steps were performed by processing the filtered supernatants using a 300-kDa cut-off tangential flow filtration system (Pall Corporation, Port Washington, NY, USA) and concentrating the samples to a final volume of 10 mL.

To remove proteins and small molecules, size exclusion chromatography was performed using a qEV automatic fraction collector (Izon Science, Christchurch, New Zealand) and a qEV 10/35 nm column (Izon Science), following the manufacturer’s protocol, to obtain sEVs with high yield and purity. The sEV fractions were concentrated using a 3-kDa molecular weight cut-off Macrosep (Pall Corporation) at 5,000 ×*g* for 20 min and stored at − 80 °C. For human serum (HS)-derived sEVs (HS sEVs), the sample was diluted in 1× phosphate-buffered saline (PBS) to a final volume of 50 mL and subjected to subsequent analyses.

### Nanoparticle tracking analysis (NTA)

The size and concentration of the sEVs were measured using a MONO ZetaView system (PMX-130; Particle Metrix, Bavaria, Germany). Calibration was performed using 100-nm polystyrene beads (Applied Microspheres, Leusden, Netherlands) diluted in a 1:250,000 ratio in deionized water. sEV samples were diluted in 1× PBS to a concentration of 4 × 10^7^ to 6 × 10^7^ particles/mL and measured under the following conditions: brightness, 30; sensitivity, 80; and temperature, 23 °C. Deionized water and PBS were filtered through a 0.1-µm syringe filter membrane (Sigma-Aldrich, St. Louis, MO, USA) before use.

### Cryogenic-transmission electron microscopy (cryo-TEM)

The size and morphology of the HT29 and HS sEVs were validated using cryo-TEM. Carbon-coated copper grids with holes (200-mesh; Quantifoil, Großlöbichau, Germany) were rendered hydrophilic by subjecting the grids to a glow discharge treatment. Subsequently, 3 µL of sEV sample was loaded and vitrified by plunging the grids into liquid ethane using a Vitrobot (Thermo Fisher Scientific). Cryo-TEM images were acquired using a JEM-2100PLUS electron microscope (JEOL, Tokyo, Japan) equipped with a complementary metal oxide semiconductor camera. The temperature was maintained at − 180 °C using liquid nitrogen.

### Western blotting

Equivalent volumes of HT29 and HS sEVs were concentrated using a qEV Concentration Kit (Izon Science) according to the manufacturer’s protocol. The concentrated sEV pellets were resuspended in PBS and 5× sodium dodecyl sulfate-polyacrylamide gel electrophoresis (SDS-PAGE) loading buffer (Biosesang, Seongnam, Republic of Korea), followed by heat denaturation at 95 °C for 10 min. The concentrated sEV samples were subjected to SDS-PAGE using a 10% TGX Stain-Free Protein Gel (Bio-Rad Laboratories, Hercules, CA, USA) at 300 V for 20 min. The resolved proteins were transferred onto a 0.2-µm polyvinylidene fluoride membrane (Bio-Rad Laboratories) at 25 V for 4 min. The membrane was washed with distilled water and blocked at room temperature for 1 h in TBST (1× TBS with 0.1% Tween-20) containing 5% bovine serum albumin (BSA). Subsequently, the membrane was incubated at room temperature for 1 h with the following primary antibodies: rabbit anti-Hsp90α (#A5006; ABclonal, Woburn, MA, USA), rabbit anti-calnexin (#A4846; ABclonal), and mouse anti-CD63 (#sc-365604; Santa Cruz, Dallas, TX, USA). All antibodies were diluted at a 1:1,000 ratio in Can Get Signal Solution 1 (Toyobo, Osaka, Japan). After washing three times with TBST, the membrane was incubated at room temperature for 45 min with the following secondary antibodies: horseradish peroxidase (HRP) goat anti-rabbit IgG (#AS014, ABclonal) and HRP goat anti-mouse IgG (#AS003, ABclonal), which were diluted (1:10,000) in Can Get Signal Solution 2 (Toyobo). After washing four times with TBST, the membrane was incubated with Clarity Western ECL Substrate (Bio-Rad Laboratories) for 5 min at room temperature, and the protein bands were visualized using the ChemiDoc Imaging system (Bio-Rad Laboratories).

### Enzymatic digestion sEV-SELEX (EDGE-SELEX)

The oligonucleotides used in the EDGE-SELEX process were synthesized by Integrated DNA Technologies (Coralville, IA, USA; Additional file 1: Table S1). The random DNA library contained primer binding regions at both ends to facilitate PCR amplification, and the central region comprised N40. HT29 sEVs were used for positive selection, whereas HS sEVs were used for counter selection. At the beginning of each round, the ssDNA library (100 nM) was diluted in binding buffer (BB; DPBS with 5 mM MgCl_2_), denatured at 95 °C for 5 min, and rapidly cooled on ice for 10 min. Initially, negative selection was performed by incubating the ssDNA library in a 1.5-mL protein low-binding microtube (Sarstedt) for 1 h to remove ssDNA that was non-specifically bound to the tube. Unbound ssDNA was collected for further selection steps.

For positive selection, the ssDNA library and HT29 sEVs were used at concentrations corresponding to the stringency of each cycle (Additional file 1: Table S2) and incubated at 37 °C. Following incubation, Exo I and T5 exo were added to remove the unbound ssDNA library from the target sEVs. The enzymes were inactivated by adding 11 µM of EDTA and heating at 80 °C for 20 min. Subsequently, the bound ssDNA library was recovered and concentrated using the Nucleospin Gel and PCR Clean-up kit (Macherey-Nagel, Düren, Germany) according to the manufacturer’s protocol.

For counter selection, the ssDNA library and HS sEVs were used at concentrations corresponding to the stringency of each and incubated at 37 °C. Following incubation, the unbound ssDNA was collected using centrifugation at 5,000 ×*g* for 5 min using a 300-kDa Nanosep ultrafiltration filter (Pall Corporation). The filtered unbound ssDNA was recovered and concentrated using a Nucleospin Gel and PCR Clean-up kit (Macherey-Nagel) according to the manufacturer’s protocol.

### Asymmetric PCR (Asy-PCR)

Asy-PCR was conducted to amplify the ssDNA library using 1.25 units of nPfu-forte DNA polymerase (Enzynomics, Daejeon, Republic of Korea), 1× nPfu buffer, forward and reverse primers at a 20:1 ratio (2 µM:100 nM), and 0.2 mM dNTPs. The initial denaturation step was performed at 95 °C for 3 min, followed by 20 cycles of denaturation at 95 °C for 10 s, annealing and extension at 68 °C for 1 min, and final extension at 68 °C for 5 min. Following Asy-PCR, the products were subjected to electrophoresis on a 2.5% agarose gel at 135 V for 40 min, and gel bands corresponding to ssDNA were extracted using a Nucleospin Gel and PCR Clean-up kit (Macherey-Nagel) according to the manufacturer’s protocol.

### Real-time PCR (qPCR) analysis

qPCR analysis was performed to confirm enzymatic digestion and monitor the EDGE-SELEX cycles. The reaction mixture contained 2 µL of 10× TOPreal qPCR premix (Enzynomics), 1 µL of 100 nM ssDNA samples either enzymatically digested or recovered from each cycle, 1 µL each of 500 nM forward and reverse primers, and distilled water at a final volume of 20 µL. An initial denaturation step was performed at 95 °C for 15 min, followed by 25 cycles of denaturation at 95 °C for 10 s, annealing at 60 °C for 15 s, and extension at 72 °C for 15 s. Subsequently, melting curve analysis was performed by gradually increasing the temperature from 55 °C to 95 °C in the CFX96 Real-Time PCR system (Bio-Rad Laboratories).

### Next-generation sequencing (NGS)

The ssDNA pool obtained from the 15th EDGE-SELEX cycle was amplified using PCR to generate double-stranded DNA (dsDNA) with the nPfu-Forte enzyme (Enzynomics), according to the manufacturer’s protocol. The product was subjected to electrophoresis on a 2.5% agarose gel. The obtained dsDNA bands were purified using the Nucleospin Gel and PCR Clean-up kit. NGS was performed using the Clinomics (Ulsan, Republic of Korea) software. Purified dsDNA was fragmented, and the adapters were ligated to both ends using the MGI FS DNA Library Prep Set (MGI, Shenzhen, China), according to the manufacturer’s protocol. Sequencing was performed on the DNBSEQ-T7 (MGI) platform with a paired-end read length of 150 bp. Sequencing data quality was assessed using FastQC (v0.11.8).

### Enzyme-linked oligonucleotide assay (ELONA)

Each well of a 96-Maxi-binding immunoplate (SPL Life Science, Pocheon, Republic of Korea) was coated with 1 × 10^8^ sEV particles and incubated at 37 °C for 2 h, followed by washing three times with 300 µL of PBST (1× PBS containing 0.1% v/v Tween-20). The wells were blocked with 300 µL of PBST containing 3% BSA at 37 °C for 2 h, followed by three washes with 300 µL of PBST. The wells were then incubated with 250 nM of 5ʹ-biotinylated aptamers at 37 °C for 1 h to enable the interaction with sEVs. Excess unbound aptamers were removed by washing three times with 300 µL of PBST. Then, 100 µL of streptavidin-HRP polymer (Sigma-Aldrich), which was diluted in a 1:1,000 ratio in PBST, was added to each well and incubated at 37 °C for 1 h. The wells were again washed three times with PBST. Subsequently, 100 µL of 1× TMB substrate solution (Invitrogen, Carlsbad, CA, USA) was added to each well and incubated at 37 °C in the dark for 5 min. The HRP-catalyzed reaction was terminated by adding 0.5 M sulfuric acid, and the absorbance was measured at 450 nm using a Spectramax iD5 plate reader (Molecular Devices, San Jose, CA, USA). The normalized ΔA was calculated as follows: ΔA = A_t_ – A_c_, where A_t_ indicates the absorbance of the sample containing both sEVs and aptamer, and A_c_ represents the absorbance of the control containing only sEVs. The sequence and concentration of the aptamers varied depending on the experiment, and all experiments were performed in triplicate.

### Determination of aptamer dissociation constant (*K*_*d*_)

The binding affinities of the aptamers were evaluated using the ELONA method by calculating the *K*_*d*_. Each well of a 96-Maxi-binding immunoplate was coated with 1 × 10^8^ sEV particles and various concentrations (0–250 nM) of 5ʹ-biotinylated aptamers (H7F-3 and H15F). The *K*_*d*_ was determined using the following equation: Y = (B_max_ × X)/(*K*_*d*_ + X), where Y indicates ΔA, B_max_ indicates maximum ΔA, and X indicates aptamer concentration.

### Topological investigation of aptamer targets on sEVs

HT29 sEVs were incubated on ice for 20 min in RIPA buffer (Biosesang) supplemented with Halt Protease Inhibitor Cocktail (Thermo Fisher Scientific) to obtain lysed sEVs. Proteinase K (ProK) treatment was conducted by incubating the sEVs with ProK (20 µg/mL; Enzynomics) at 37 °C for 1 h, followed by incubation with phenylmethylsulfonyl fluoride (5 mM) at 25 °C for 10 min to inactivate the ProK. sEVs were then incubated with trypsin (0.25%; Welgene) at 37 °C for 30 min. Trypsin was then inactivated by adding an equal volume of PBS containing 10% v/v FBS. All treated samples were stored at − 80 °C until further use. The subsequent steps were performed using the same protocol as that described above.

### Aptamer-based loop-mediated isothermal amplification for sEV detection (ABLE) system

To perform the ABLE system, 1 × 10^10^ HT29 sEV particles were incubated with 50 nM H7F-3 aptamer in 1× BB at 37 °C for 30 min. The sample was isolated and purified using an Izon qEV with a 35 nm GEN 2 column (Izon Science) according to the manufacturer’s protocol. The concentration was measured using NTA. Ligation was performed by incubating 2 µL of the sample with 2 µL of 10× T4 ligation buffer, 1 µL each of 2 nM L- and R-loop probes (Additional file 1: Table S3), 0.5 µL of T4 ligase (Enzynomics), and 13.5 µL of diethylpyrocarbonate (DEPC)-treated water at 37 °C for 30 min. Subsequently, 2 µL of the ligation sample was mixed with 10 µL of WarmStart Colorimetric LAMP 2× Master mix with UDG (New England Biolabs, Ipswich, MA, USA), 0.4 µL each of 20 µM forward and backward inner primers (Additional file 1: Table S3), and 0.4 µL of loop-mediated isothermal amplification (LAMP) fluorescent dye (New England Biolabs); the total volume was adjusted to 20 µL using DEPC-treated water. After the LAMP reaction proceeded at 65 °C for 1 h in the CFX96 Real-Time PCR system (Bio-Rad Laboratories), the products were analyzed using electrophoresis on a 2.5% agarose gel at 135 V for 30 min. The gel images were obtained using the ChemiDoc Imaging system (Bio-Rad Laboratories).

### Denaturing polyacrylamide gel electrophoresis

To confirm the ligation of the L- and R-loop probes (Additional file 1: Table S3) through the presence of aptamers, each sample was mixed with 2× Novex TBE-urea sample buffer (Invitrogen) and heated at 95 °C for 10 min. Electrophoresis was then performed on a 14% denaturing urea polyacrylamide gel. After pre-running the gel at 200 V for 40 min, the samples were loaded onto the gel and run at 150 V for 40 min. Finally, the gel was stained in the dark with GreenStar Nucleic Acid Staining Solution (Bioneer, Daejeon, Republic of Korea) for 10 min and imaged using a ChemiDoc Imaging system (Bio-Rad Laboratories).

### Statistical analysis

Statistical analyses were conducted using the GraphPad Prism 8 software (GraphPad Inc., La Jolla, CA, USA). All data are presented as the mean ± standard deviation. The two-tailed Student’s *t*-test was used to determine the significance of differences between sample pairs. Significance was set at *p* < 0.01, which was represented as **; *p* > 0.05 was represented as non-significant (ns). Each experiment was performed in triplicate to ensure robustness.

## Results and discussion

### Design of enzymatic digestion sEV-SELEX (EDGE-SELEX)

**Fig. 1:**
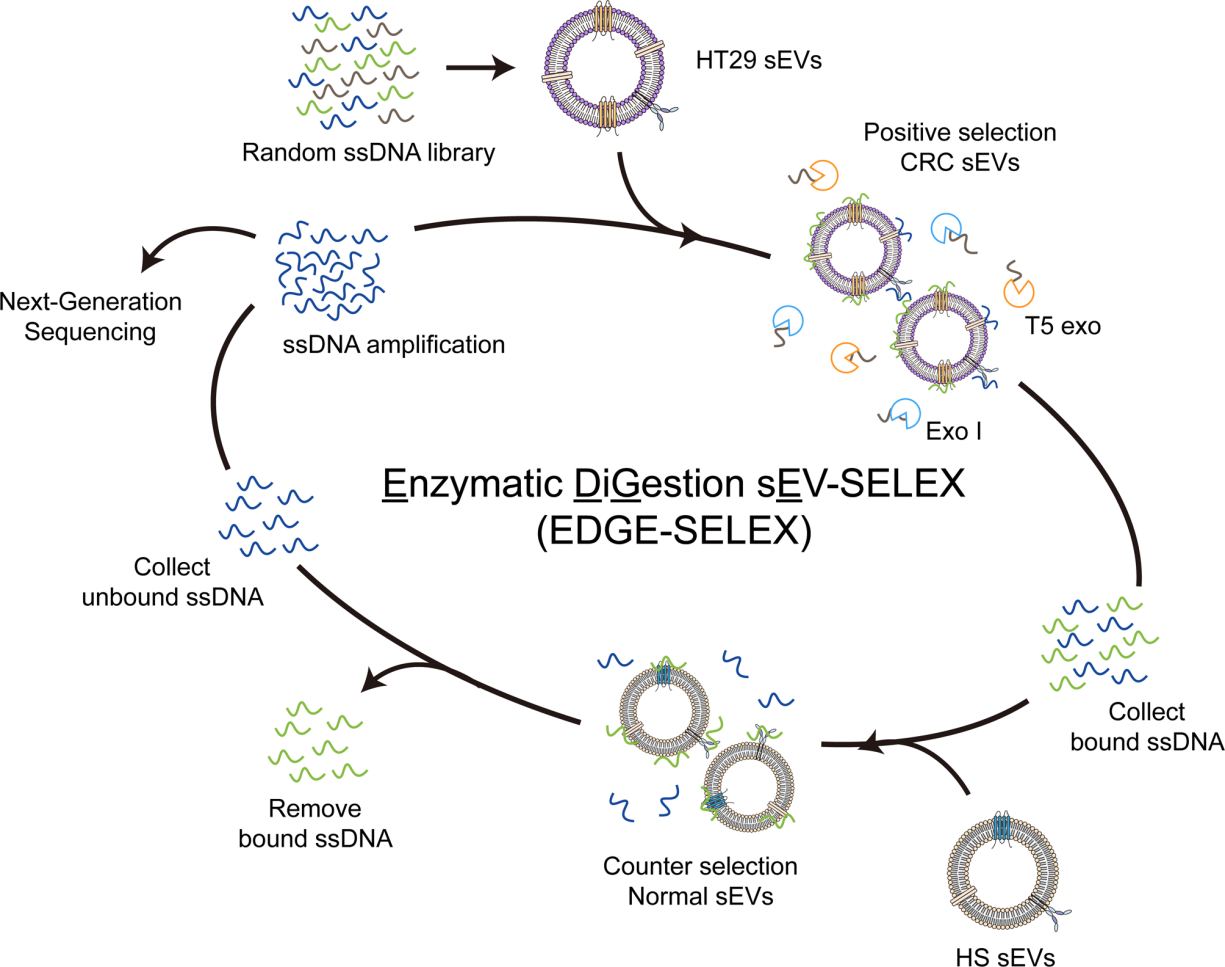
Illustration of the enzymatic digestion sEV-systematic evolution of ligands by exponential enrichment (EDGE-SELEX) system. Workflow of the EDGE-SELEX cycle. CRC, colorectal cancer; sEV, small extracellular vesicle; Exo I, exonuclease I; T5 exo, T5 exonuclease; and HS, human serum

We proposed the EDGE-SELEX system to screen aptamers specific to sEVs, designed to preserve the three-dimensional environment without requiring immobilization (Fig. 1). The key component of the proposed system is the use of two enzymes: (i) Exo I, which degrades ssDNA in the 3ʹ to 5ʹ direction, and (ii) T5 exo, which exerts both exonuclease activity on dsDNA in the 5ʹ to 3ʹ direction and ssDNA endonuclease activity. We postulated that Exo I and T5 exo were expected to be effective only when the substrate ssDNA or dsDNA was unbound from the target sEVs. This would enable the separation of bound and unbound aptamer libraries without immobilization onto a solid support, such as an immunoplate. We selected the CRC cell line, HT29, and HS sEVs as positive and counter-targets, respectively. The EDGE-SELEX cycles were repeated under increasingly stringent conditions until no further increase in enrichment of ssDNA was observed (Additional file 1: Table S2).

First, we evaluated whether Exo I and T5 exo can effectively digest unbound ssDNA libraries by performing qPCR and native PAGE analyses. Both Exo I and T5 exo showed efficient cleavage of random ssDNA libraries, as evidenced by the increased Cq values and disappearance of gel bands corresponding to the ssDNA libraries (Additional file 2: Figure S1). Additionally, we determined the optimal enzyme concentrations to be 2 U/µL for Exo I and 4 U/µL for T5 exo. Moreover, the co-presence of Exo I and T5 exo was more effective in digesting the unbound ssDNA libraries than Exo I or T5 exo alone (Additional file 2: Figure S2). Subsequently, we characterized both HT29 and HS sEVs using NTA, cryo-TEM, and western blotting to validate the isolated sEVs. The vesicles exhibited an average size of approximately 100 nm, and typical sEV biomarkers, such as CD63 and HSP90α, were detected, whereas non-sEV biomarkers, such as Calnexin, were not observed (Additional file 2: Figure S3). These results support the integrity of the sEVs used in this study [37].

### In vitro selection of aptamers via EDGE-SELEX

**Fig. 2:**
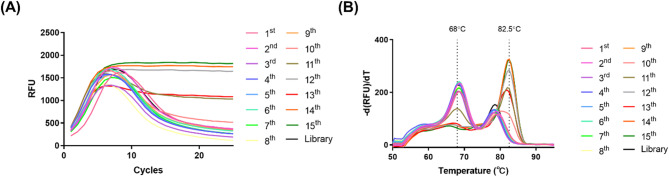
Monitoring the EDGE-SELEX cycle via qPCR analysis. (**A**) qPCR amplification curve analysis of EDGE-SELEX cycles. (**B**) Melting curve analysis of the EDGE-SELEX cycles

During the EDGE-SELEX process, we monitored the variation in the heterogeneity of the ssDNA library using qPCR and melting curve analyses. As presented in Fig. 2, heterogeneous random ssDNA libraries typically exhibited an amplification pattern in which the degree of amplification initially increased, reached a peak, and subsequently decreased. This pattern differed from the general qPCR amplification curve [20, 38]. This phenomenon has been attributed to the ssDNA library having identical primer binding sites but different sequences in the central 40 random nucleotides (N40) region, leading to incomplete dsDNA hybridization and a decrease in the SYBR green fluorescence signal intensity [39]. The same pattern was observed in the melting curve analysis, where a high peak was observed in the lower melting temperature (Tm; 68 °C) for the random ssDNA library [40].

As the SELEX cycles progressed, ssDNAs that bound specifically to the positive-target HT29 sEVs became enriched over cycles, whereas non-binding ssDNA levels gradually decreased. The decrease in heterogeneity of the ssDNA library indicates an increase in homogeneity, which is reflected by the plateau following the peak in the qPCR amplification curve and a major peak at a higher Tm (82.5 °C) in the melting curve. Based on these results, we stopped EDGE-SELEX at the 15th cycle and subjected the ssDNA library from that cycle to NGS.

### Assessment and post-SELEX optimization of aptamer candidates

**Fig. 3:**
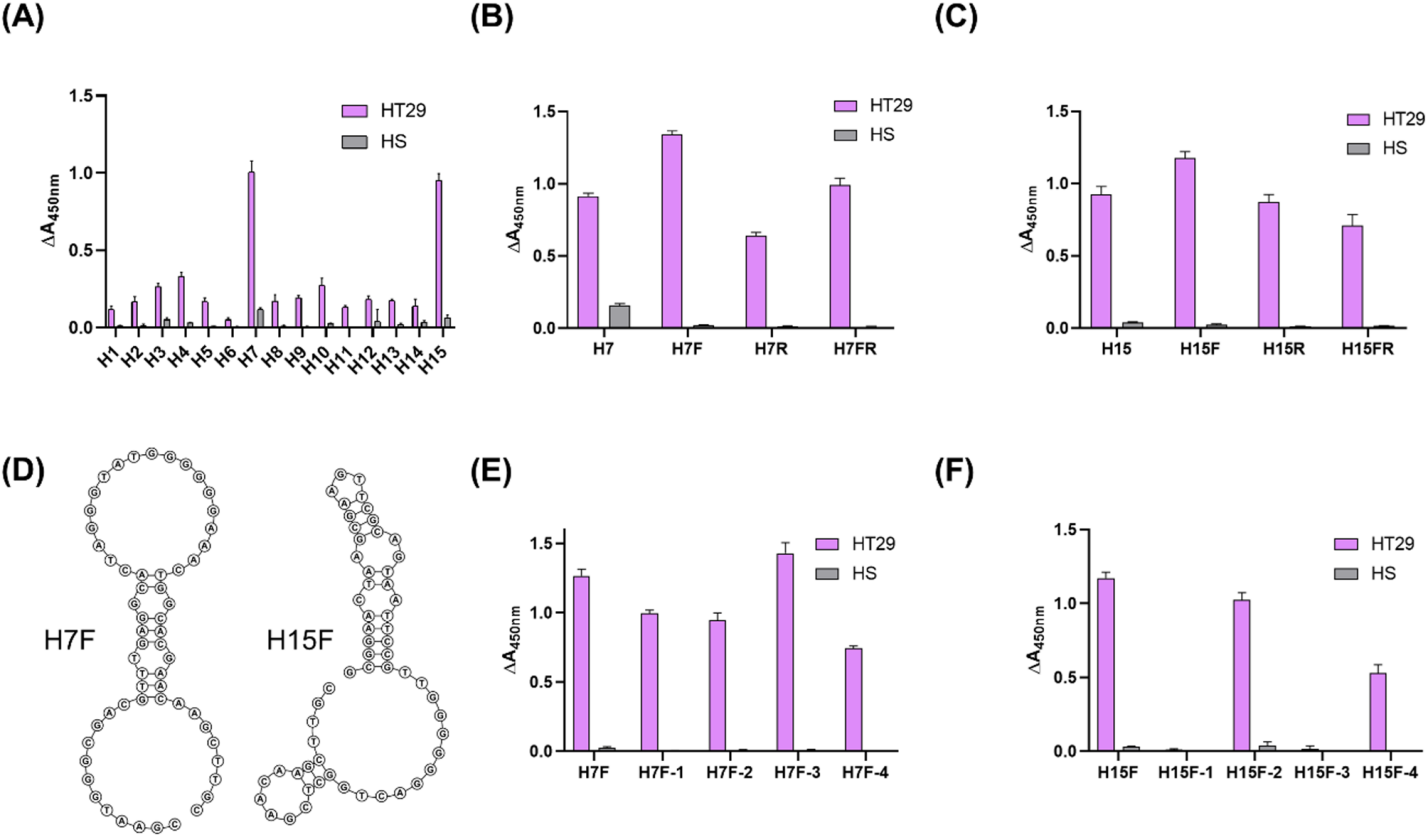
Selection and post-SELEX optimization of the aptamer candidates. (**A**) ELONA assessment of the top 15 aptamer candidates identified using EDGE-SELEX. (**B**, **C**) Assessment of the binding affinities of the H7 and H15 aptamers and their variants [deleted binding sites: F, forward; R, reverse; and FR, both forward and reverse] via ELONA. (**D**) Predicted 2D structures of the selected H7F and H15F aptamers. (**E**,** F**) Assessment of the binding affinities of the H7F and H15F aptamers and their variants (−1, −2, −3, and −4) via ELONA. ELONA, enzyme-linked oligonucleotide assay; HS, human serum

As summarized in Additional file 1: Table S4 and Additional file 2: Figure S4, we selected the top 15 aptamer candidates ranked by frequency (%) based on NGS analysis and evaluated their binding affinities to HT29 sEVs using ELONA. As depicted in Fig. 3A, the H7 and H15 aptamers exhibited higher ELONA signal intensity in the presence of the positive-target HT29 sEVs than in the presence of the counter-target HS sEVs. Subsequently, we performed post-SELEX optimization using aptamers H7 and H15 to identify the aptamers with improved binding affinities. The ssDNA library used in EDGE-SELEX contains primer binding sites that could either positively or negatively influence the binding affinity of the aptamer to the target [30]. Therefore, we prepared truncated variants of the H7 and H15 aptamers by removing the forward (F), reverse (R), or both primer binding sites (FR) (Additional file 1: Table S5; Additional file 2: Figure S5 and S6). The removal of the forward primer binding site was associated with an enhanced binding affinity to the target HT29 sEVs for both the H7 and H15 aptamers (Fig. 3B and C). These results suggest that the forward primer binding site may negatively influence binding affinity; therefore, we selected H7F and H15F for further post-SELEX optimization.

We analyzed the secondary structures of the H7F and H15F aptamers (Fig. 3D) and identified a symmetrical hairpin structure in the H7F aptamer and two hairpin structures in the H15F aptamer. Hairpins are known to play a crucial role in aptamer binding [41]; therefore, we generated truncated variants for H7F (H7F-1, H7F-2, H7F-3, and H7F-4) and H15F (H15F-1, H15F-2, H15F-3, and H15F-4) while maintaining the hairpin structures (Additional file 2: Figure S7). Among the H7F variants, H7F-3, in which the single-stranded overhang sequence at both ends was eliminated, exhibited the highest binding affinity to HT29 sEVs. However, the binding affinity of H7F-4 further decreased after the shortening of the H7F-3 stem region (Fig. 3E). For H15F, the binding affinities were higher for H15F-2 and H15F-4, where the ssDNA linking the two hairpin structures was intact, than in H15F-1 and H15F-3, where only the individual hairpin was present (Fig. 3F). These results suggest that evaluating non-canonical regions may also contribute to aptamer binding and could be considered during the post-selection process. However, H15F, which was not further truncated, exhibited the highest binding affinity among the tested variants. Therefore, we selected H7F-3 and H15F as the optimal aptamer sequences for subsequent experiments.

### Characterization of H7F-3 and H15F aptamers

**Fig. 4:**
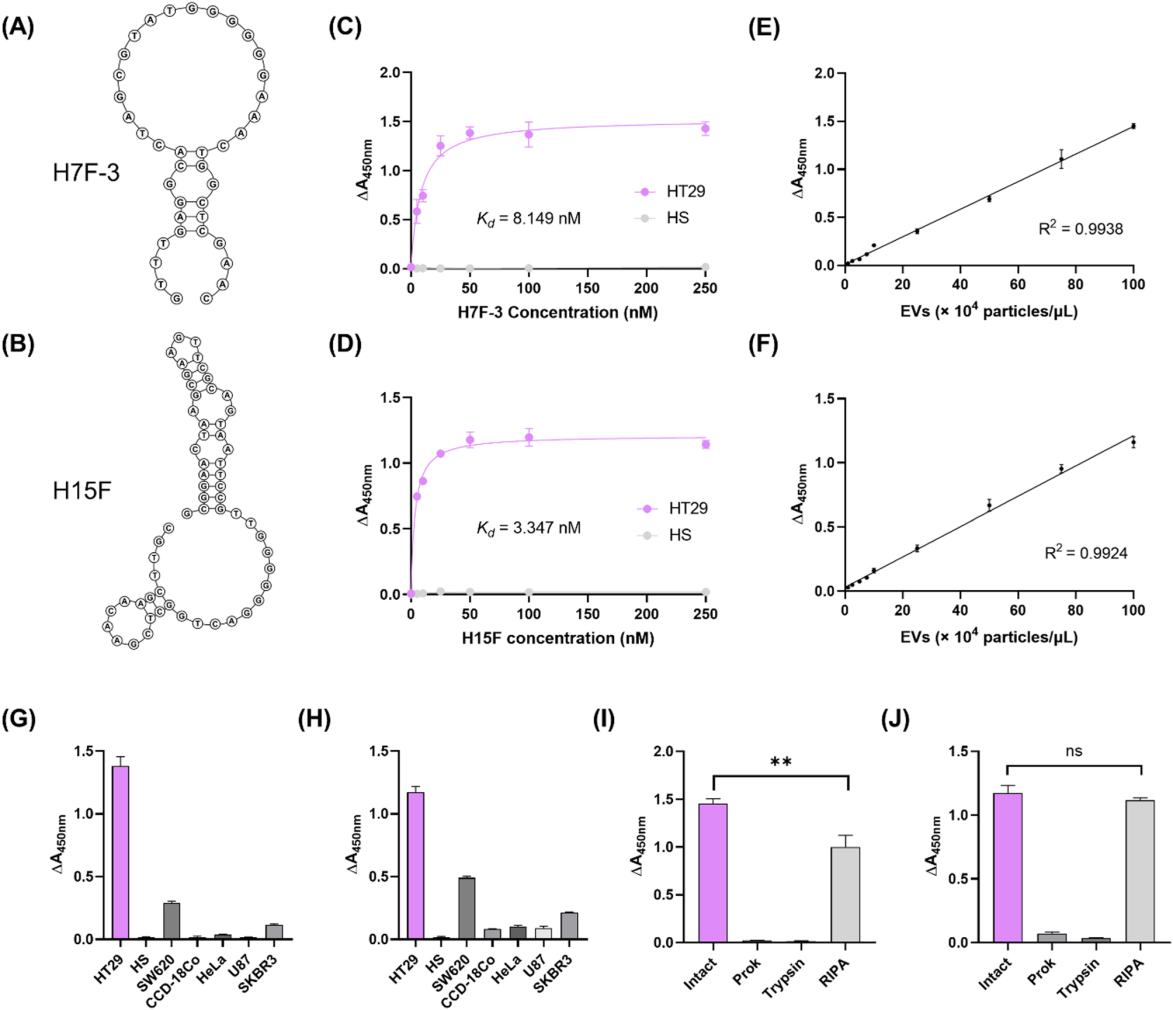
Characterization of the H7F-3 and H15F aptamers. (**A**,** B**) Predicted 2D structures of the H7F-3 and H15F aptamers. (**C**,** D**) Determination of the dissociation constant (*K*_*d*_) values of the H7F-3 and H15F aptamers using ELONA. The Y axis indicates the absorbance of various concentrations (0–250 nM) of the H7F-3 and H15F aptamers towards HT29 and HS sEVs. (**E**,** F**) Sensitivity of the H7F-3 and H15F aptamers to HT29 sEVs. (**G**,** H**) Specificity of the H7F-3 and H15F aptamers. (**I**,** J**) Topological validation of biomarkers targeted by the H7F-3 and H15F aptamers. All absorbance values were obtained using ELONA. ELONA, enzyme-linked oligonucleotide assay; HS, human serum; sEVs, small extracellular vesicles; and ProK, proteinase K. Two-tailed *t*-test: **p < 0.01, non-significant (ns) *p* > 0.05

To characterize the selected aptamers (Fig. 4A and B), we first assessed the *K*_*d*_ by varying H7F-3 and H15F aptamer concentrations via the ELONA method. The ELONA signal intensities for both H7F-3 and H15F increased as aptamer concentrations increased, until the signal from the positive-target HT29 sEVs reached saturation (Fig. 4C and D). H7F-3 and H15F had *K*_*d*_ values of 8.149 nM and 3.347 nM, respectively, which are comparable to previously reported aptamers targeting sEVs [20, 30]. In contrast, no signal increase was observed with the counter-target HS even at high aptamer concentrations, and the *K*_*d*_ could not be determined.

We subsequently validated the detection performance of the aptamers by performing sensitivity and specificity assessments using ELONA. To assess the sensitivity, we performed a linear regression analysis over the range of 1 × 10^4^–1 × 10^6^ sEV particles/µL. As presented in Fig. 4E and F, H7F-3 followed the equation “y = 0.01435x + 0.01058” with an estimated limit of detection (LOD) of 1.03 × 10^3^ particles/µL, whereas H15F followed the equation “y = 0.01181x + 0.03082,” with an estimated LOD of 1.25 × 10^3^ sEV particles/µL. These results indicate sensitivity comparable to previously reported aptamer-based detection strategies for cancer-derived sEVs [20, 30]. Furthermore, we evaluated the specificity of the aptamers against sEVs derived from various cell lines that were not included as counter-targets during the EDGE-SELEX process. The aptamers exhibited a negligible ELONA signal in normal colon (CCD-18Co), cervical cancer (HeLa), and glioblastoma (U-87 MG) cells, similar to the response for HS. However, slightly increased signals were observed for CRC (SW620) and breast cancer (SKBR3) cells (Fig. 4G and H). These results suggest that the biomarker in HT29 sEVs targeted by the aptamers may be present at low levels in SW620 and SKBR3 cells.

Finally, we investigated the topological location of the biomarkers recognized by the aptamers by treating the sEVs with ProK, trypsin, and RIPA buffer and analyzing them using ELONA (Fig. 4I and J). For both aptamers, ELONA signals were absent in samples treated with ProK or trypsin, suggesting that the target biomarker protein is located on the sEV surface. However, different effects were observed between the two aptamers in samples treated with RIPA buffers. Specifically, the ELONA signal from H7F-3 was lower under RIPA-treated conditions than under intact conditions, whereas no significant difference in signal intensity for H15F. These results suggest that H15F may recognize an epitope of the target protein that is either anchored in the phospholipid bilayer membrane or present in its isolated state. In contrast, H7F-3 appeared to preferentially bind to the target protein in its intact state, integrated within the phospholipid bilayer.

These results suggest that the conformational state of the target protein in sEVs could influence the binding affinity of the aptamers. Furthermore, these findings are consistent with the intended advantages of EDGE-SELEX in maintaining the native state of sEVs and support the importance of preserving the physiological properties of target biomacromolecules during the SELEX process.

### Design and validation of H7F-3 and H15F aptamer-based applications

**Fig. 5:**
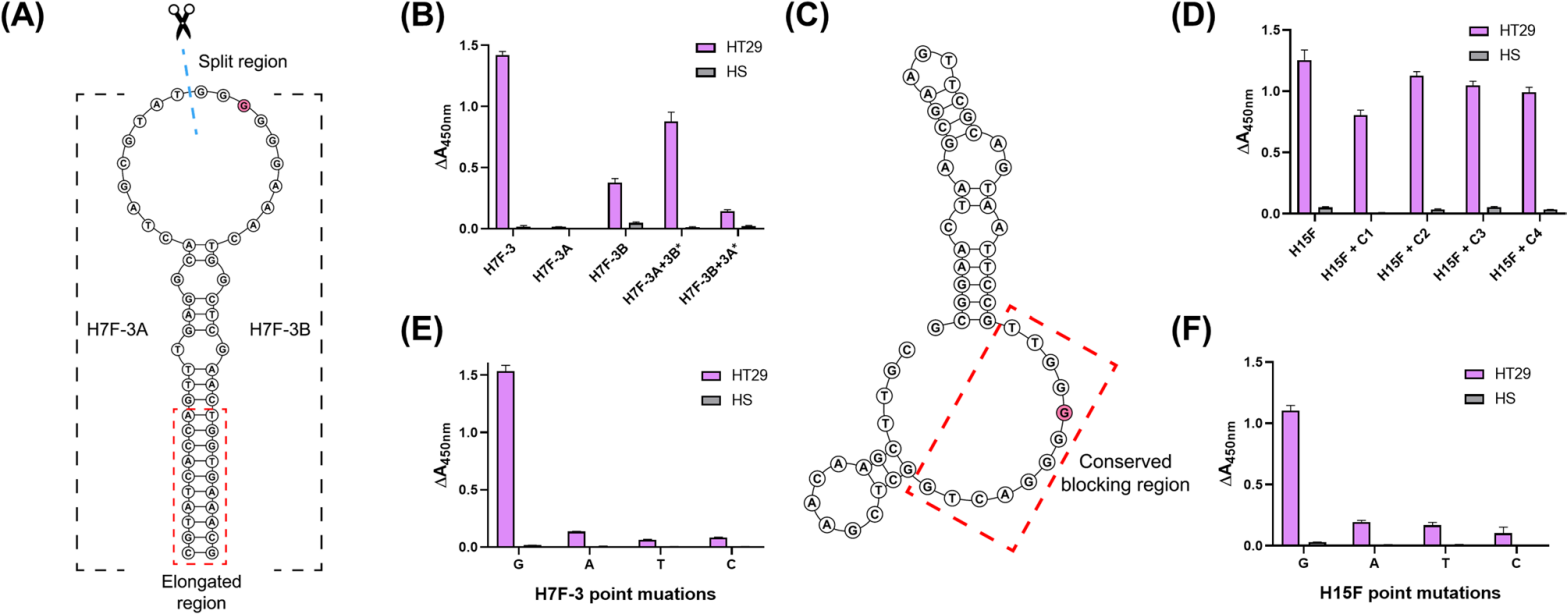
Design and feasibility of the application of the H7F-3 and H15F aptamers. (**A**) Design of split strategy using the H7F-3 aptamer. The red box indicates the elongated region. The nucleotide indicated in pink represents a point mutation site. (**B**) Assessment of the binding affinities of the split aptamer strands via ELONA. The asterisk (*) indicates the non-biotinylated, split aptamer strands. (**C**) Design of blocking strategy using the H15F aptamer. The red box indicates the conserved blocking region. The nucleotides highlighted in pink represent a point mutation site. (**D**) Feasibility of blocking strategy using the H15F aptamer. Biotinylated H15F aptamer hybridized with various blocker probes (C1, C2, C3, and C4). (**E**) Effects of point mutation on the G6 motif in the H7F-3 aptamer. (**F**) Effects of point mutation on the G6 motif in the H15F aptamer. ELONA, enzyme-linked oligonucleotide assay; HS, human serum

We designed various strategies for diagnostic applications using H7F-3 and H15F aptamers based on their predicted secondary structures. For H7F-3, we splitted the core of the hairpin and extended the opposite ends by leveraging its symmetrical structure, named H7F-3A and H7F-3B, respectively (Additional file 1: Table S6; Fig. 5A). We then assessed both split aptamer strands (H7F-3A and H7F-3B) to determine whether they assembled to function together in the presence of the target sEVs. First, each strand was biotinylated at the 5ʹ ends, and the binding affinities were evaluated using the ELONA method. In Fig. 5B, H7F-3A did not produce an ELONA signal, whereas H7F-3B generated a low signal. The use of the biotinylated H7F-3A and non-biotinylated H7F-3B (H7F-3B*) partially restored the ELONA signal. In contrast, the combination of non-biotinylated H7F-3A (H7F-3A*) and biotinylated H7F-3B caused a reduction of the signal intensity. This result may be attributed to the biotinylated region located in the core of the hairpin structure. Additionally, we investigated the ability of the split aptamer strands to bind to target sEVs by changing the length of the elongated region. We gradually shortened the elongated region of the split aptamer strands by two nucleotides (nt), followed by evaluation using ELONA and PAGE (Additional file 2: Figure S8). After reducing the length of the elongation region by 4 nt (–4*), the ELONA signal intensity started to decrease in the presence of the target sEVs. In contrast, it significantly decreased when the length was reduced by 6, 8, and 10 nt (–6*, − 8*, and −10*, respectively). These results suggest the importance of the elongation region in the split H7F-3 aptamer for its potential use as a detection probe.

For the H15F aptamer, given that the ssDNA sequence linking the two hairpin structures was found to be important (Fig. 3F), we designed various blocker DNAs (C1, C2, C3, and C4; Additional file 2: Figure S9A) complementary to the H15F aptamer (Additional file 1: Table S6; Fig. 5C). All blocker DNAs hybridized with H15F exhibited higher ELONA signals than those obtained with H15F alone, except for C1, which was fully complementary to H15F (Additional file 2: Figure S9B; Fig. 5D). These results support the potential use of H15F aptamers and blocker DNAs as a structure-switching detection strategy for target sEVs.

### Identification and validation of the G6 motif critical for aptamer binding

While analyzing the sequence and structure of H7F-3 and H15F, we identified a common G6 sequence motif in which six consecutive G residues were located in the hairpin loop region of H7F-3 and the single-stranded region of H15F. Additionally, among the top 15 candidate aptamers, only H7 and H15 contained the G6 motif, suggesting that this motif may contribute to binding to the target sEVs (Additional file 2: Figure S10). To assess the effect of the G6 motif on binding affinity, we performed a point mutation experiment based on the opposite rationale of the aptamer truncation study. Specifically, we mutated the third position from the 5ʹ end within the central region of the G6 motif (highlighted in pink in Fig. 5A and C; Additional file 1: Table S7), since truncation had been performed from the terminal region, which was expected to have minimal effect on binding. Mutations in both aptamers significantly reduced the ELONA signal intensity, suggesting that the intact G6 motif plays an important role in aptamer binding to its target HT29 sEVs (Fig. 5E and F). This motif could provide important insights into nucleic acid-protein interactions, similar to the G-quadruplex structures in AS1411-nucleolin binding, CpG sites recognized by toll-like receptor 9, and protospacer adjacent motif sequence in the CRISPR-Cas system [42–44]. Furthermore, the G6 motif has the potential to be incorporated into the design of SELEX random libraries to develop motif-guided advanced SELEX strategies.

### Establishment of an aptamer-based sEV detection system

**Fig. 6:**
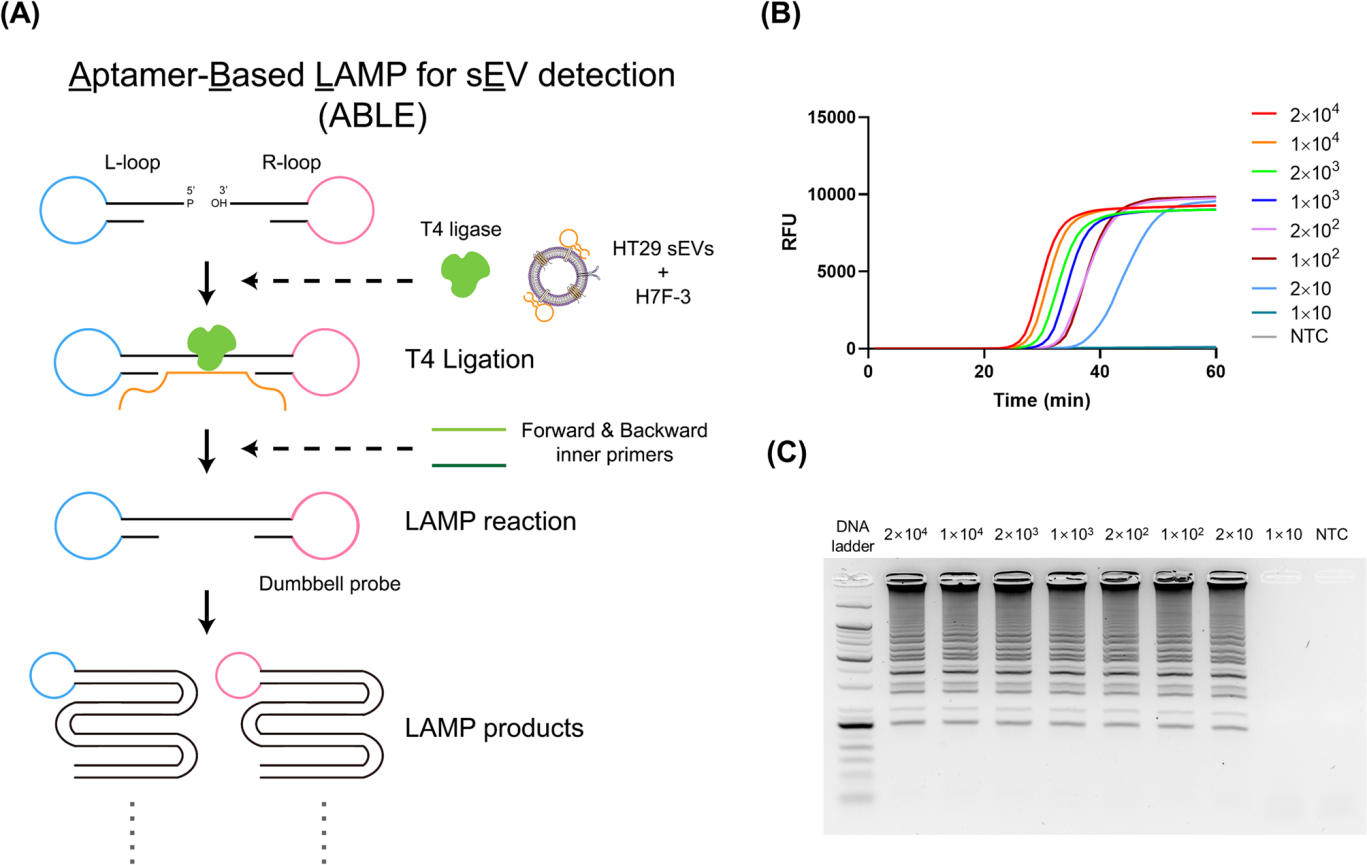
Aptamer-based loop-mediated isothermal amplification (LAMP) for sEV detection (ABLE) system. (**A**) Illustration of the ABLE system. (**B**) Sensitivity of the ABLE system via real-time fluorescence monitoring. (**C**) Sensitivity of the ABLE system via agarose gel electrophoresis. NTC, non-target control; sEV, small extracellular vesicle

We constructed an aptamer-based detection system for sEVs using H7F-3, which is shorter (39-mer) than H15F (59-mer) and showed high binding affinity to HT29 sEVs. As described in Fig. 6A, we applied the LAMP, which is an isothermal nucleic acid amplification method for the detection of target sEVs, and termed it the ABLE system. Specifically, we designed a ligation reaction to occur only in the presence of H7F-3 aptamers bound to the target sEVs, which were subsequently integrated into the LAMP assay. Thus, in a sample where H7F-3 aptamers were bound to the sEVs, two hairpin probes (L- and R-loops) with sequences complementary to the aptamer were ligated via a T4 ligase-catalyzed reaction. The ligated dumbbell probes are then amplified through LAMP using two inner primers.

We confirmed the formation of the ligated dumbbell probe with the aptamers using denaturing PAGE analysis. The two hairpin probes were ligated and generated ligation products with the highest molecular weight, as evidenced in the lane containing the H7F-3 aptamers, all hairpin probes, and T4 ligase (Additional file 2: Figure S11A). We then optimized the T4 ligase concentration (10, 20, 30, 40, and 50 U/µL) and ligation time (15, 30, 45, 60, 90, and 120 min). The ligated dumbbell probe was formed under various conditions, and the optimal conditions for T4 ligase concentration and ligation time were determined to be 10 U/µL and 30 min, respectively (Additional file 2: Figure S11B and S11C). Furthermore, we optimized the concentration of the hairpin probes for LAMP. The amplification time at 10 pM was significantly delayed in the presence of the H7F-3 aptamer, whereas at 1 nM, non-specific amplification signals began to appear at 50 min even in the absence of the H7F-3 aptamer (Additional file 2: Figure S12). Thus, we selected 100 pM as the optimal hairpin probe concentration, as it allowed relatively rapid detection of the target sEVs without non-specific amplification.

Under optimized conditions, we evaluated the sensitivity of the ABLE system using HT29 sEVs at different concentrations ranging from 10 to 2 × 10^4^ particles/µL via real-time fluorescence monitoring and agarose gel electrophoresis (Fig. 6B and C). As depicted in Fig. 6B, a high fluorescence signal, proportional to LAMP products, was observed in the presence of HT29 sEVs, even at concentrations as low as 20 particles/µL. This signal was clearly distinguishable from that of the non-target control (NTC) for the 60 min of the LAMP reaction. Previously reported concentration of plasma sEVs in CRC patients are approximately 10^9^ particles/mL (10^6^ particles/µL) [45]. Tumor-derived sEV subpopulations have been reported to account for 0.01 to 3% of total plasma sEVs [19]. Based on these estimates, the sensitivity of the ABLE system appears sufficient to detect clinically relevant levels of CRC-derived sEVs.

The ABLE system operates under isothermal conditions, making it suitable for point-of-care testing (POCT). It may be adapted for portable devices that utilize isothermal amplification technologies such as LAMP or recombinase polymerase amplification (RPA) [46–48]. Moreover, the system could be integrated into microfluidic isothermal platforms and combined with lateral flow assay (LFA) or colorimetric readouts commonly used with LAMP, thereby broadening its applicability for POCT [49, 50]. Nevertheless, several challenges remain for clinical applications. First, validation with sEV samples derived from CRC patients is necessary to confirm the clinical effectiveness of the identified aptamers and the ABLE system, as sEVs in clinical samples can be affected by factors such as protein corona formation on their surfaces [51, 52]. Second, further studies are required to achieve multidisciplinary integration, including microfluidics and high-throughput sample processing platforms. These advancements would enable rapid and efficient analysis of large patient cohorts and facilitate the development of a practical POCT system.

## Conclusion

In the present study, we developed EDGE-SELEX, a SELEX technology designed to preserve the intact three-dimensional structure of sEVs without immobilization, thereby reflecting their native physiological state in a clinical environment. Using EDGE-SELEX, we identified H7F-3 and H15F aptamers targeting sEVs derived from the representative CRC cell line, HT29. These aptamers demonstrated high binding affinities for membrane protein biomarkers of sEVs. Notably, H7F-3 aptamer exhibited a stronger binding affinity toward intact proteins anchored in the membrane of sEVs than to the isolated state. Structural analysis further revealed their potential diagnostic applications, which include the split and blocking strategies for H7F-3 and H15F aptamers, respectively. We also identified the presence of the G6 motif in the aptamers, which appears to play an important role in their binding to target sEVs. Leveraging the high binding abilities and relatively short sequence length of H7F-3 aptamer (39-mer), we established the ABLE system, enabling the sensitive detection of HT29 sEVs at concentrations as low as 20 particles/µL. These findings underscore the significance of the EDGE-SELEX technology in preserving the native, non-immobilized state of sEVs. Given its isothermal feature, the ABLE system holds promise for POCT applications. Nevertheless, further validation with clinical samples and multidisciplinary optimization are required to realize its potential and enable its translation into routine clinical use.

## Supplementary Information

Supplementary Material 1

Supplementary Material 2

## Data Availability

No datasets were generated or analysed during the current study.

## Abbreviations

EVs: Extracellular vesicles
sEVs: Small extracellular vesicles
ssDNA: Single-stranded DNA
SELEX: Systematic evolution of ligands by exponential enrichment
EDGE-SELEX: Enzymatic digestion sEV-systematic evolution of ligands by exponential enrichment
Exo I: Exonuclease I
T5 exo: T5 exonuclease
CRC: Colorectal cancer
HT29 sEVs: HT29-dervied sEVs
LAMP: Loop-mediated isothermal amplification
ABLE: Aptamer-based loop-mediated isothermal amplification for sEV detection
PBS: Phosphate-buffered saline
FBS: Fetal bovine serum
HS: Human serum
HS sEVs: Human serum-derived sEVs
Cryo-TEM: Cryogenic-transmission electron microscopy
SDS-PAGE: Sodium dodecyl sulfate-polyacrylamide gel electrophoresis
BSA: Bovine serum albumin
BB: Binding buffer
Asy-PCR: Asymmetric PCR
qPCR: Real-time PCR
K_d_: Dissociation constant
ProK: Proteinase K
NGS: Next-generation sequencing
NTA: Nanoparticle tracking analysis
dsDNA: Double-stranded DNA
ELONA: Enzyme-linked oligonucleotide assay
HRP: Horseradish peroxidase
NTC: Non-target control
RIPA: Radio-immunoprecipitation assay
DEPC: Diethyl pyrocarbonate
LOD: Limit of detection
POCT: Point-of-care testing
RPA: Recombinase polymerase amplification
