# Supplementary material for: Immobilization-free SELEX for aptamer discovery targeting colorectal cancer-derived small extracellular vesicles
Source: J Nanobiotechnology. 2025 Nov 25;23:738. doi: 10.1186/s12951-025-03813-0 (PMC12648788; doi:10.1186/s12951-025-03813-0)
Supplement: Supplementary file 1 — Supplementary Material 1 [file 12951_2025_3813_MOESM1_ESM.docx]

**Additional file 1: Supplementary Tables**

**Immobilization-free SELEX for aptamer discovery targeting colorectal cancer-derived small extracellular vesicles**

Eun Sung Lee^a,1^, Byung Seok Cha^a,1^, Junhyeong Kim^a^, Seung Hyeon Reo^a^, Jinseo Son^a^, and Ki Soo Park^a,b,^*

^a^ Department of Biological Engineering, College of Engineering, Konkuk University, Seoul 05029, Republic of Korea

^b^ Advanced Materials Program, Department of Biological Engineering, Konkuk University, Seoul 05029, Republic of Korea

^1^ These authors contributed equally to this study.

* To whom correspondence should be addressed: Tel +82-2-450-3742; Fax: +82-2-450-3742; E-Mail: [akdong486@konkuk.ac.kr](mailto:akdong486@konkuk.ac.kr)

This file contains Tables S1–S7.

**Table S1.** Oligonucleotides used in the EDGE-SELEX process.

|  | Sequence (5′–3′) |
| --- | --- |
| Random library | ATCCAGAGTGACGCAGCA-*N40*-CTGGCTCGAACAAGCTTGC |
| Forward primer | ATCCAGAGTGACGCAGCA |
| Reverse primer | GCAAGCTTGTTCGAGCCAG |

**Table S2.** The stringent conditions used in the EDGE-SELEX process.

| **Cycle** | **HT29 sEVs**  **(Particles)** | **HS sEVs**  **(Particles)** | **Incubation time**  **(Positive)** | **Enzyme incubation time**  **(Exo I & T5 exo)** | **Incubation time**  **(Counter)** |
| --- | --- | --- | --- | --- | --- |
| 1 | 1 × 10^9^ | - | 60 min | 15 min | - |
| 2 |  |  |  |  |  |
| 3 |  |  |  |  |  |
| 4 | - | 1 × 10^9^ | - | - | 60 min |
| 5 | 5 × 10^8^ | - | 45 min | 30 min | - |
| 6 |  |  |  |  |  |
| 7 |  |  |  |  |  |
| 8 | - | 2 × 10^9^ | - | - | 120 min |
| 9 | 2.5 × 10^8^ | - | 30 min | 45 min | - |
| 10 |  |  |  |  |  |
| 11 |  |  |  |  |  |
| 12 | - | 4 × 10^9^ | - | - | 180 min |
| 13 | 1 × 10^8^ | - | 20 min | 60 min | - |
| 14 |  |  |  |  |  |
| 15 |  |  |  |  |  |

Exo I, exonuclease I; HS, human serum; sEV, small extracellular vesicle; T5 exo, T5 exonuclease.

**Table S3.** Oligonucleotide sequences used in the ABLE system.

|  | Sequence (5′-3′) | |
| --- | --- | --- |
| L-loop | | Phosphate-ATACCCTAGTGCCTCATCGGACGTTTATGGGGATGGCCAACGCAGTTGATCAGTCCGCAGCACGTCAAACCGTATGTCCCATCCCCATAAACGTCCGAT |
| R-loop | | GCTCCTCCACACGCTTCCATTGTATCCACCGTAGCCAGTCTTAAGGTGGGCTGCGTGGTGATGGAAGCGTGTGGAGGAGCAGCCAGTTTCCCCCC |
| FIP | | ATCGGACGTTTATGGGGATGGGACATACGGTTTGACGTGCTG |
| BIP | | GCTCCTCCACACGCTTCCATTGTATCCACCGTAGCCAGTC |

L, R, FIP, and BIP represent left, right, forward inner primer, and backward inner primer, respectively. ABLE, aptamer-based loop-mediated isothermal amplification for sEV detection.

**Table S4.** Sequences of the top 15 aptamer candidates obtained using EDGE-SELEX.

|  | Sequence (5′-3′) | Read | Frequency  (%) |
| --- | --- | --- | --- |
| H1 | ACAAAACGAGGACACTAATGTATCTTCACAAGCGCCATCG | 547664 | 10.77 |
| H2 | CGATGGCGCTTGTGAAGATACATTAGTGTCCTCGTTTTGT | 514959 | 10.13 |
| H3 | TCTAACGATTTCCCCAGTATACTACCTCTCACGGCGCCCG | 188434 | 3.71 |
| H4 | TCCACGCCTCCTATGCTCTCTCTGCGCTCTCCTCTCCAAG | 182355 | 3.59 |
| H5 | CGGGCGCCGTGAGAGGTAGTATACTGGGGAAATCGTTAGA | 175344 | 3.45 |
| H6 | CTTGGAGAGGAGAGCGCAGAGAGAGCATAGGAGGCGTGGA | 159260 | 3.13 |
| H7 | CGAATGGGCGACGTTTGAGGCACTAGGGTATGGGGGGAAA | 153711 | 3.02 |
| H8 | AGCGGAATGGCGCAGTGAGTGCTATGGGGTATCGCTTAAC | 150513 | 2.96 |
| H9 | GTTAAGCGATACCCCATAGCACTCACTGCGCCATTCCGCT | 150301 | 2.96 |
| H10 | TCCCCCCAACGGAATTACTGCGAACTTCGCTTAGTTCCGC | 143024 | 2.81 |
| H11 | CCTCAACCCCACGAAAAAGCGGCCTTCGTGACCTCTACA | 139224 | 2.74 |
| H12 | TGTAGAGGTCACGAAGGCCGCTTTTTCGTGGGGTTGAGG | 110873 | 2.18 |
| H13 | CCCACCCCCCTACAGTTCCCCCACCCACTCCTATCTCCCG | 86526 | 1.70 |
| H14 | AGTATTCCGCTTACATTCTCTCACAAAGCCATCCCTCCGC | 71310 | 1.40 |
| H15 | GCGGAACTAAGCGAAGTTCGCAGTAATTCCGTTGGGGGGA | 71005 | 1.40 |

The sequences of forward and reverse primer binding sites were not indicated.

**Table S5.** Oligonucleotide sequences of H7, H15, and their variants.

|  | Sequence (5′-3′) |
| --- | --- |
| H7 | ATCCAGAGTGACGCAGCACGAATGGGCGACGTTTGAGGCACTAGGGTATGGGGGGAAACTGGCTCGAACAAGCTTGC |
| H7F | CGAATGGGCGACGTTTGAGGCACTAGGGTATGGGGGGAAACTGGCTCGAACAAGCTTGC |
| H7R | ATCCAGAGTGACGCAGCACGAATGGGCGACGTTTGAGGCACTAGGGTATGGGGGGAAA |
| H7FR | CGAATGGGCGACGTTTGAGGCACTAGGGTATGGGGGGAAA |
| H7F-1 | GTTTGAGGCACTAGGGTATGGGGGGAAACTGGCTCGAACAAGCTTGC |
| H7F-2 | CGAATGGGCGACGTTTGAGGCACTAGGGTATGGGGGGAAACTGGCTCGAAC |
| H7F-3 | GTTTGAGGCACTAGGGTATGGGGGGAAACTGGCTCGAAC |
| H7F-4 | GAGGCACTAGGGTATGGGGGGAAACTGGCTC |
| H15 | ATCCAGAGTGACGCAGCAGCGGAACTAAGCGAAGTTCGCAGTAATTCCGTTGGGGGGACTGGCTCGAACAAGCTTGC |
| H15F | GCGGAACTAAGCGAAGTTCGCAGTAATTCCGTTGGGGGGACTGGCTCGAACAAGCTTGC |
| H15R | ATCCAGAGTGACGCAGCAGCGGAACTAAGCGAAGTTCGCAGTAATTCCGTTGGGGGGA |
| H15FR | GCGGAACTAAGCGAAGTTCGCAGTAATTCCGTTGGGGGGA |
| H15F-1 | GCGGAACTAAGCGAAGTTCGCAGTAATTCCG |
| H15F-2 | GCGGAACTAAGCGAAGTTCGCAGTAATTCCGTTGGGGGGACTG |
| H15F-3 | GCTCGAACAAGCTTGC |
| H15F-4 | TTGGGGGGA CTGGCTCGAACAAGCTTGC |

The sequences used for enzyme-linked oligonucleotide assay were modified at the 5ʹ terminus with biotin.

**Table S6.** Oligonucleotide sequences of H7F-3 and H15F and their variants.

|  | Sequence (5′-3′) |
| --- | --- |
| H7F-3 | GTTTGAGGCACTAGGGTATGGGGGGAAACTGGCTCGAAC |
| H7F-3A | CGTATCACCAGTTTGAGGCACTAGGGTAT |
| H7F-3B | GGGGGGAAACTGGCTCGAACTGGTGATACG |
| H7F-3B (-2) | GGGGGGAAACTGGCTCGAACTGGTGATA |
| H7F-3B (-4) | GGGGGGAAACTGGCTCGAACTGGTGA |
| H7F-3B (-6) | GGGGGGAAACTGGCTCGAACTGGT |
| H7F-3B (-8) | GGGGGGAAACTGGCTCGAACTG |
| H7F-3B (-10) | GGGGGGAAACTGGCTCGAAC |
| H15F | GCGGAACTAAGCGAAGTTCGCAGTAATTCCGTTGGGGGGACTGGCTCGAACAAGCTTGC |
| H15F C1 | GCAAGCTTGTTCGAGCCAGTCCCCCCAACGGAATTACTGCGAACTTCGCTTAGTTCCGC |
| H15F C2 | CAGTCCCCCCAACGGAA |
| H15F C3 | CGAGCCAGTCCCCCCAA |
| H15F C4 | CAGTCCCCCCAA |

The sequences used for enzyme-linked oligonucleotide assay were modified at the 5′ terminus with biotin.

**Table S7.** Oligonucleotide sequences to investigate the point mutation on the G6 motif.

|  | | Sequence (5′-3′) |
| --- | --- | --- |
| H7F-3 | GTTTGAGGCACTAGGGTATGGGGGGAAACTGGCTCGAAC | |
| H7F-3 mut A | GTTTGAGGCACTAGGGTATGGAGGGAAACTGGCTCGAAC | |
| H7F-3 mut T | GTTTGAGGCACTAGGGTATGGCGGGAAACTGGCTCGAAC | |
| H7F-3 mut G | GTTTGAGGCACTAGGGTATGGTGGGAAACTGGCTCGAAC | |
| H15F | GCGGAACTAAGCGAAGTTCGCAGTAATTCCGTTGGGGGGACTGGCTCGAACAAGCTTGC | |
| H15F mut A | GCGGAACTAAGCGAAGTTCGCAGTAATTCCGTTGGAGGGACTGGCTCGAACAAGCTTGC | |
| H15F mut T | GCGGAACTAAGCGAAGTTCGCAGTAATTCCGTTGGTGGGACTGGCTCGAACAAGCTTGC | |
| H15F mut G | GCGGAACTAAGCGAAGTTCGCAGTAATTCCGTTGGCGGGACTGGCTCGAACAAGCTTGC | |

The sequences used for enzyme-linked oligonucleotide assay were modified at the 5ʹ terminus with biotin. The red font indicates the point mutation sites.
