# Supplementary material for: Immobilization-free SELEX for aptamer discovery targeting colorectal cancer-derived small extracellular vesicles
Source: J Nanobiotechnology. 2025 Nov 25;23:738. doi: 10.1186/s12951-025-03813-0 (PMC12648788; doi:10.1186/s12951-025-03813-0)
Supplement: Supplementary file 2 — Supplementary Material 2 [file 12951_2025_3813_MOESM2_ESM.docx]

**Additional file 2: Supplementary Figures**

**Immobilization-free SELEX for aptamer discovery targeting colorectal cancer-derived small extracellular vesicles**

Eun Sung Lee^a,1^, Byung Seok Cha^a,1^, Junhyeong Kim^a^, Seung Hyeon Reo^a^, Jinseo Son^a^, and Ki Soo Park^a,b,^*

^a^ Department of Biological Engineering, College of Engineering, Konkuk University, Seoul 05029, Republic of Korea

^b^ Advanced Materials Program, Department of Biological Engineering, Konkuk University, Seoul 05029, Republic of Korea

^1^ These authors contributed equally to this study.

* To whom correspondence should be addressed: Tel +82-2-450-3742; Fax: +82-2-450-3742; E-Mail: [akdong486@konkuk.ac.kr](mailto:akdong486@konkuk.ac.kr)

This file contains Figures S1–S12.


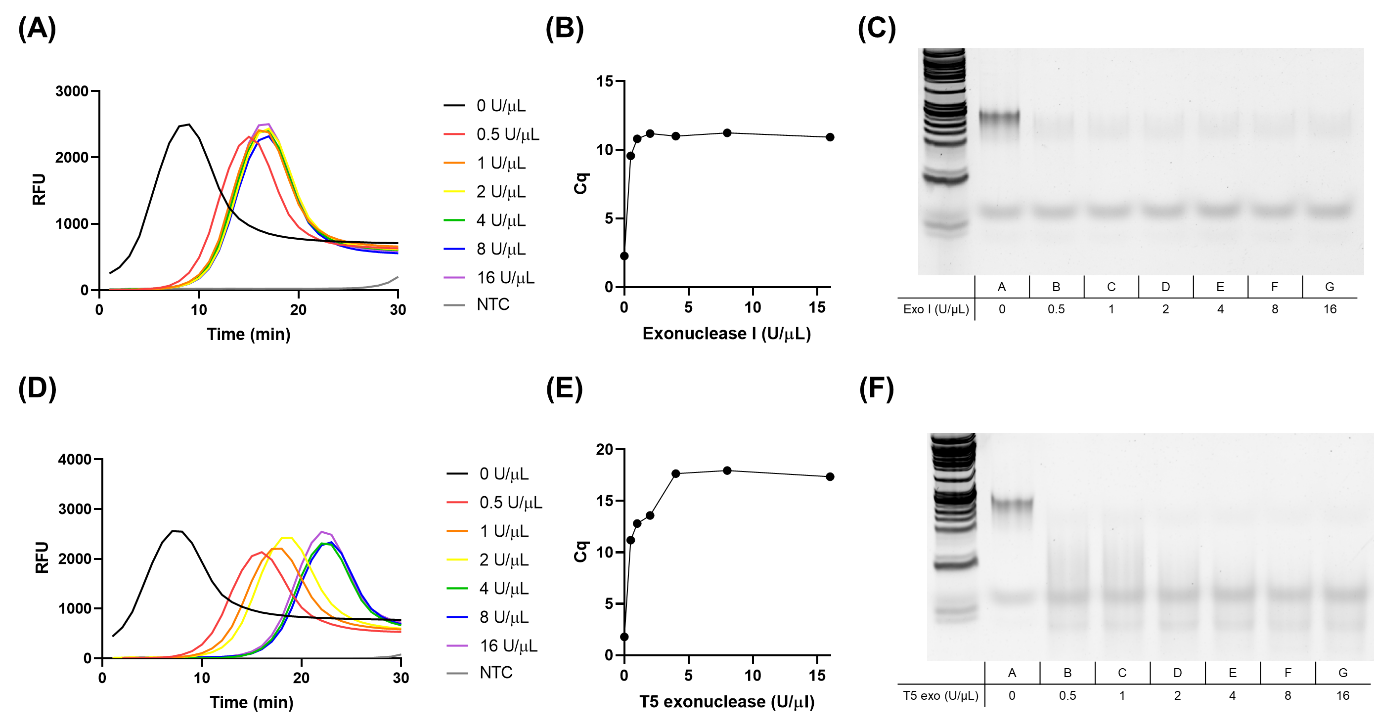


**Figure S1. Optimization of Exo I and T5 exo concentration for EDGE-SELEX.** (A) Real-time PCR (qPCR) amplification curve presenting various Exo I concentrations (0–16 U/μL) for a random single-stranded DNA (ssDNA) library. (B) Cq value for the optimization of Exo I concentrations. (C) Native polyacrylamide gel electrophoresis (PAGE) analysis with various Exo I concentrations (0–16 U/μL) for the random ssDNA library. (D) qPCR amplification curve with various T5 exo concentrations (0–16 U/μL) for the random ssDNA library. (E) Cq value for the optimization of T5 exo concentrations. (F) Native PAGE analysis with various T5 exo concentrations (0–16 U/μL) for the random ssDNA library. Exo I, exonuclease I; T5 exo, T5 exonuclease.


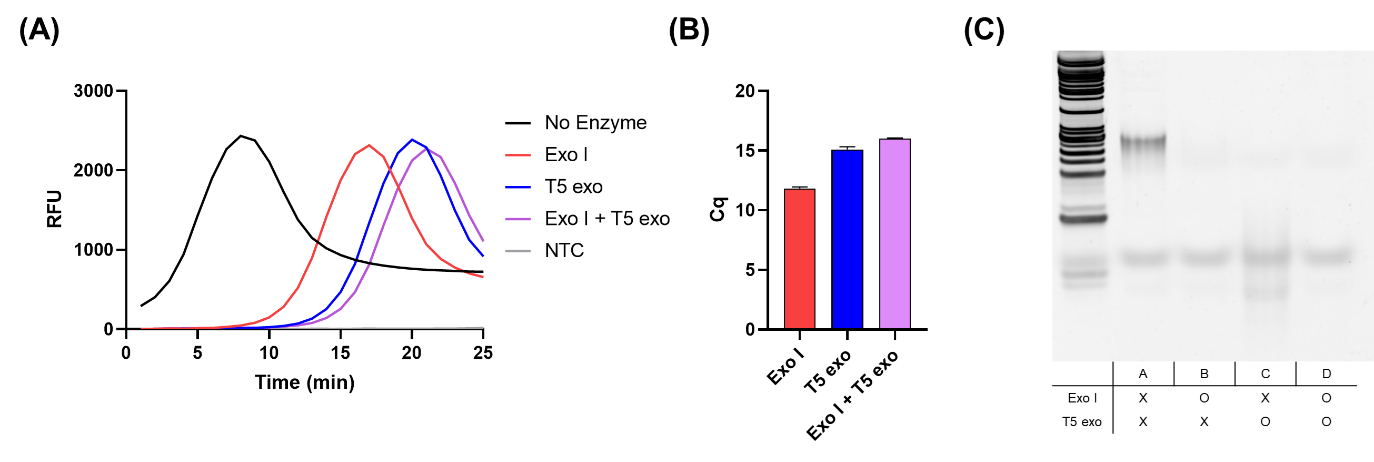


**Figure S2. Feasibility of enzyme digestion.** Comparison of enzymatic digestion for different enzyme combinations. (A) qPCR amplification curve with various enzyme combinations (Exo I only, T5 exo only, and Exo I + T5 exo) for the random single-stranded DNA (ssDNA) library. (B) Cq value of qPCR amplification curve in (A). (C) Native PAGE analysis with various enzyme combinations for a random ssDNA library. Exo I, exonuclease I; NTC, non-target control; T5 exo, T5 exonuclease.


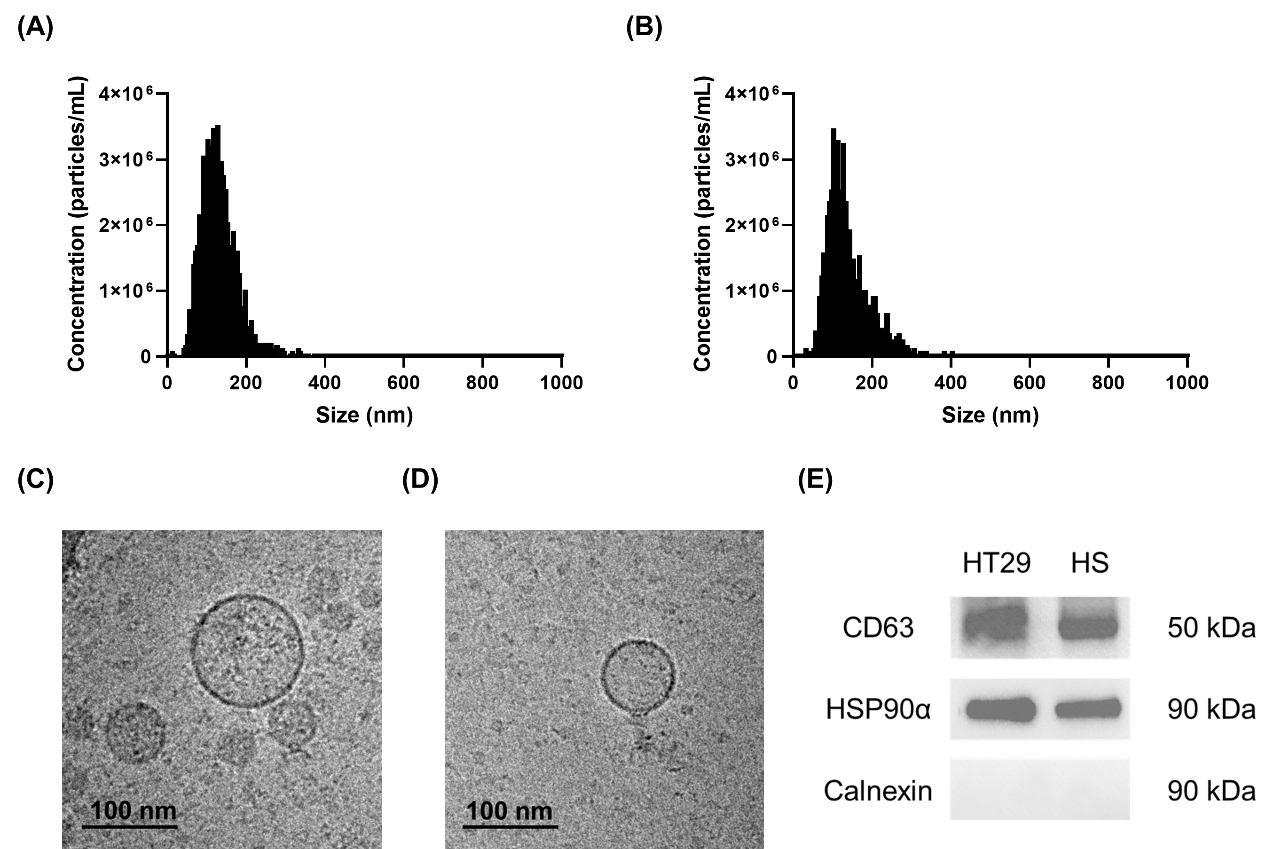


**Figure S3. Characterization of HT29 and human serum (HS) sEVs.** (A) Size distribution of HT29 sEVs. (B) Size distribution of HS sEVs. (C) Cryogenic-transmission electron microscopy image of HT29 sEVs. (D) Cryo-TEM image of HS sEVs. Scale bar: 100 nm. (E) Western blotting analysis of the expression of sEV markers in HT29 and HS sEVs. sEV, small extracellular vesicle.


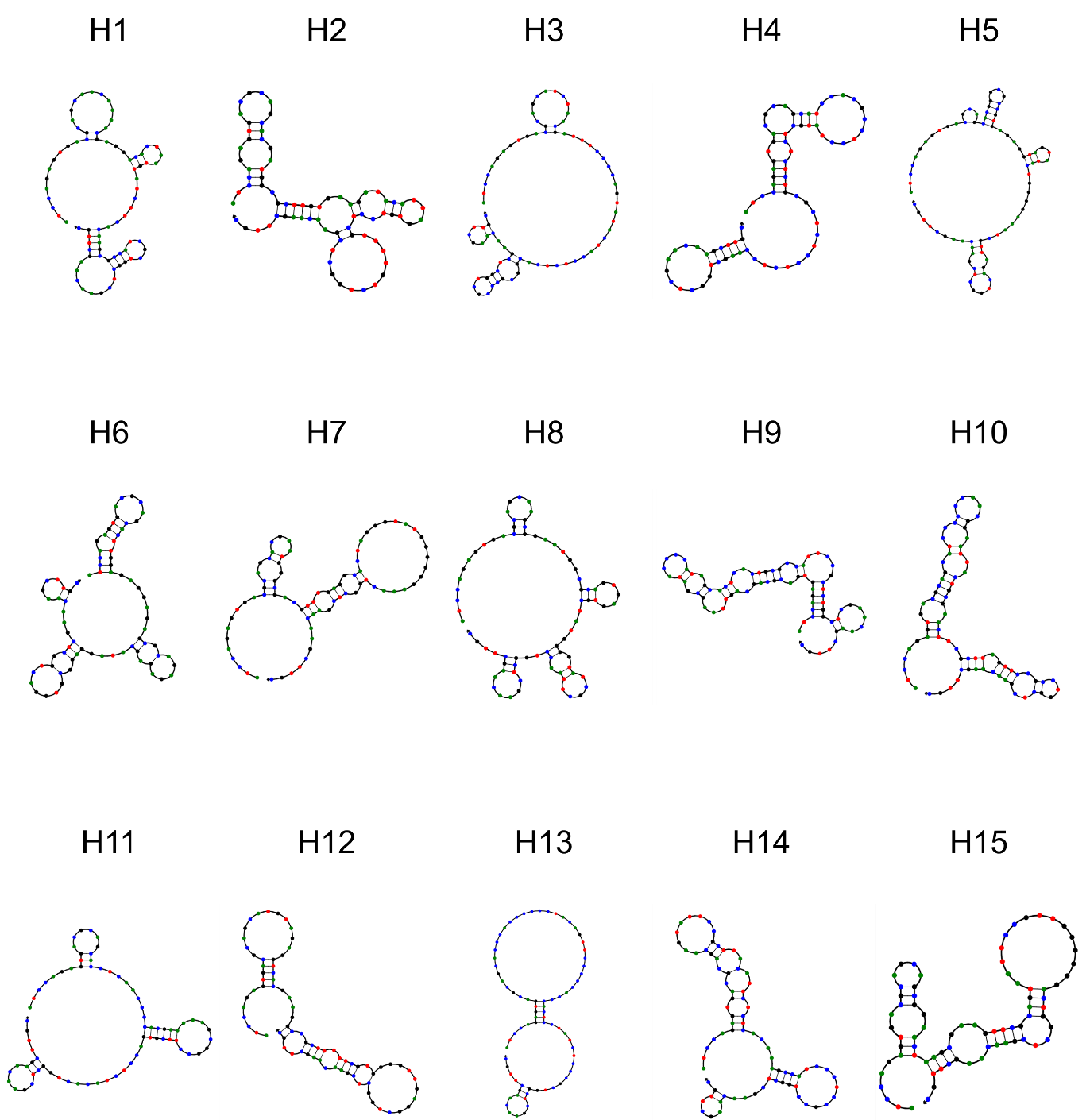


**Figure S4. Structural analysis of the top 15 aptamer candidates.** Aptamer structures were predicted using the NUPACK software. The green, blue, black, and red colors indicate adenine, cytosine, guanine, and thymine, respectively.


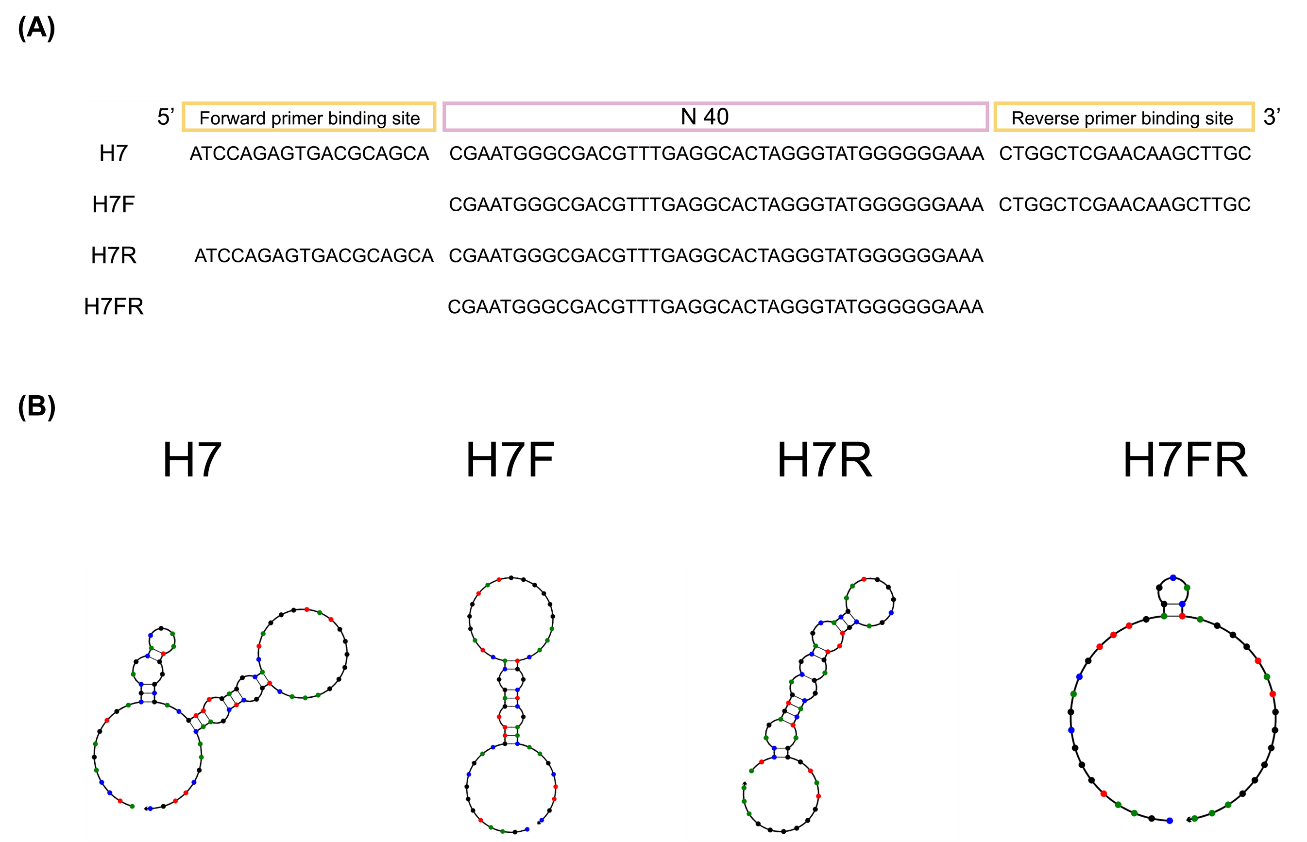


**Figure S5. Sequence information of the H7 aptamer and its variants.** (A) Sequence alignment of the H7 aptamer and its variant. (B) Predicted structure of the H7 aptamer and its variant using NUPACK software. The green, blue, black, and red colors indicate adenine, cytosine, guanine, and thymine, respectively.


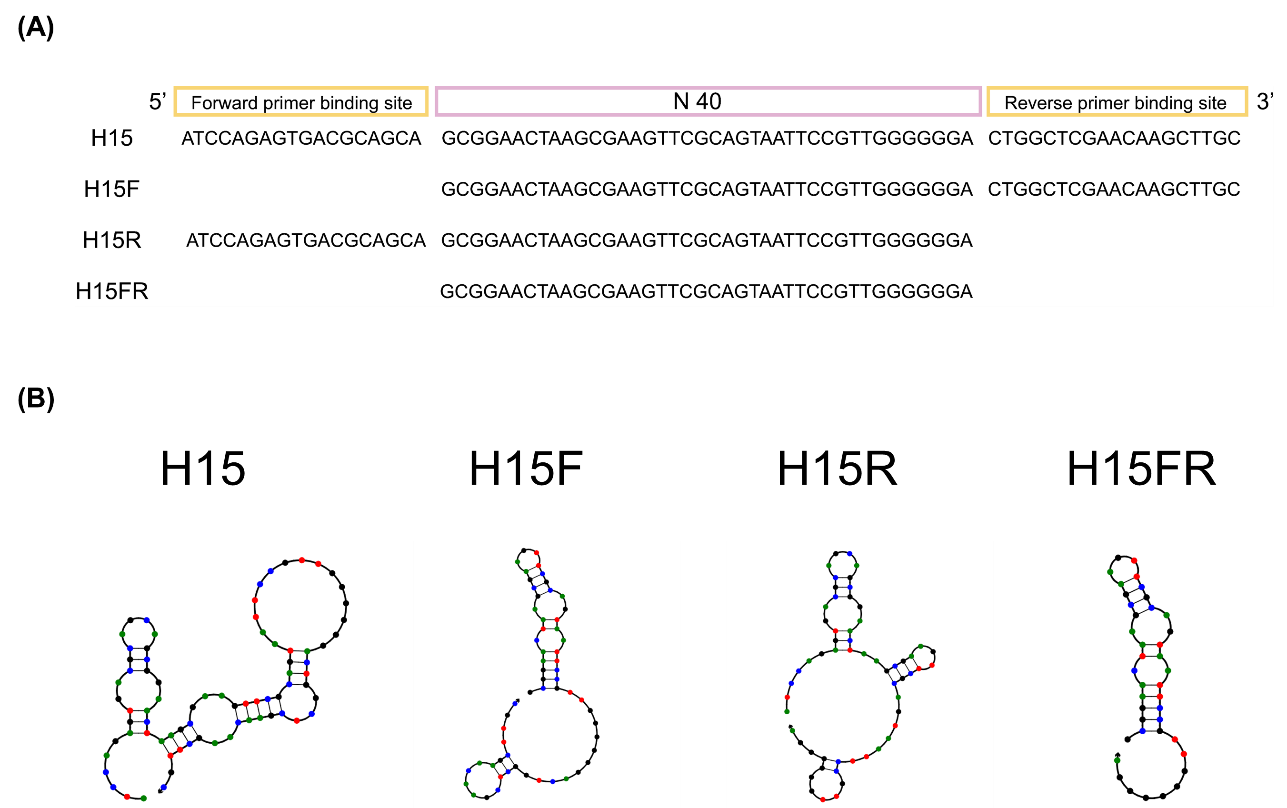


**Figure S6. Sequence information of the H15 aptamer and its variants.** (A) Sequence alignment of the H15 aptamer and its variant. (B) Predicted structure of the H15 aptamer and its variant using NUPACK software. The green, blue, black, and red colors indicate adenine, cytosine, guanine, and thymine, respectively.


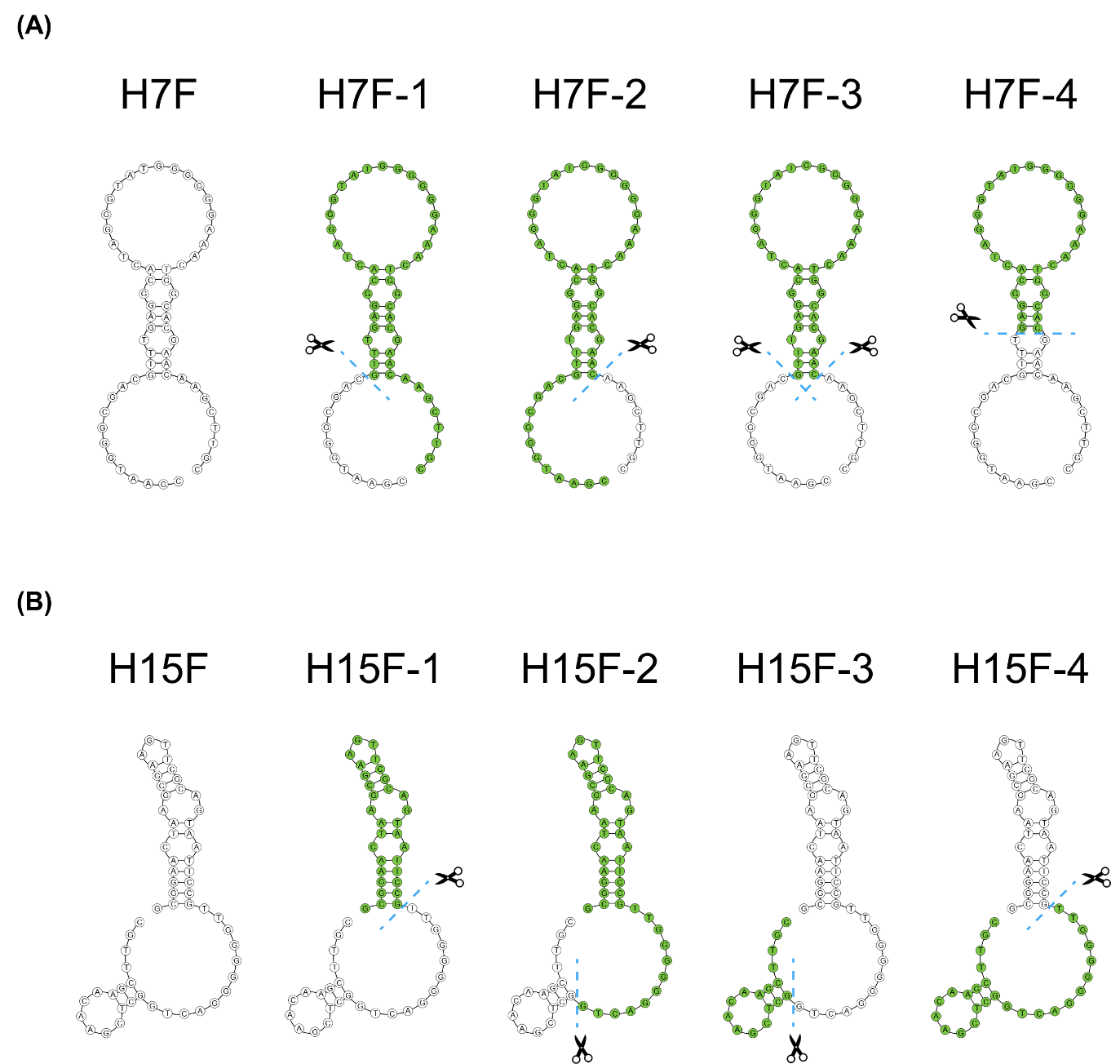


**Figure S7. Design of the H7F and H15F variants.** (A) Design of the H7F aptamer and its truncated variants (H7F-1, H7F-2, H7F-3, and H7F-4). The regions marked with scissors were truncated; all hairpin structures were preserved. (B) Design of the H15F and its truncated variants. (H15F-1, H15F-2, H15F-3, and H15F-4). The regions marked with scissors were truncated, whereas the sequences highlighted in green were preserved.


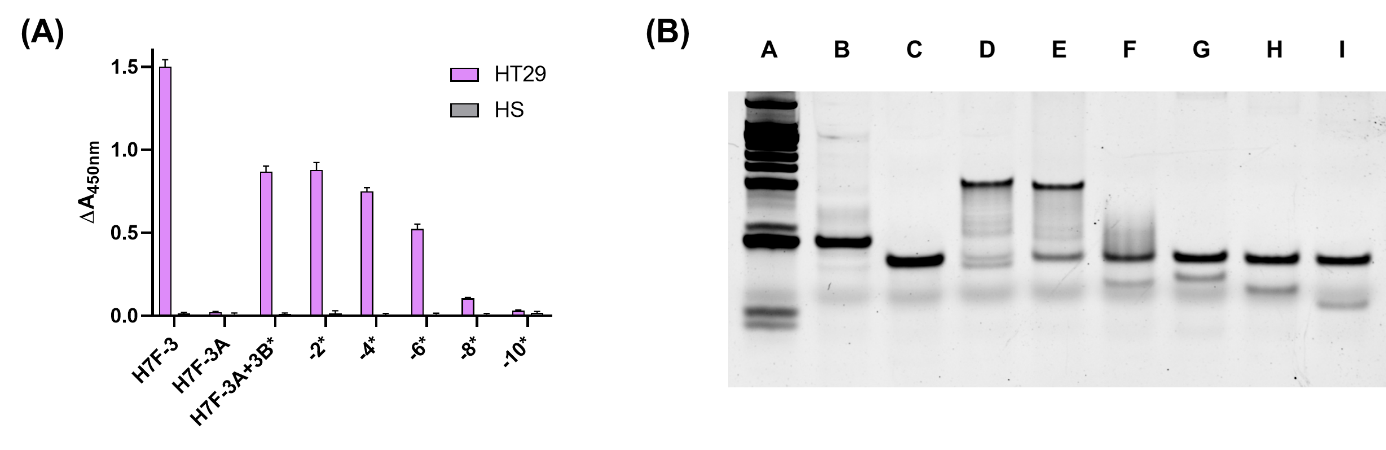


**Figure S8. Elongated region assessment of the H7F-3 split aptamer.** (A) Binding affinity of the H7F-3 split aptamer with varying lengths in the elongated region (–2, –4, –6, –8, and –10 nucleotides (nt) of H7F-3B*) via enzyme-linked oligonucleotide assay (ELONA). The “–” indicates the number of nucleotides truncated from the 3ʹ end of the elongated region of H7F-3B; the asterisk (*) indicates the non-biotinylated, split aptamer strands. (B) Native PAGE analysis to confirm the hybridization of H7F-3 split aptamer without target HT29 sEVs. Lane A: DNA ladder, Lane B: H7F-3, Lane C: H7F-3A, Lane D: H7F-3A + H7F-3B*, Lane E: H7F-3A + H7F-3B(-2)*, Lane F: H7F-3A + H7F-3B(-4)*, Lane G: H7F-3A + H7F-3B(-6)*, Lane H: H7F-3A + H7F-3B(-8)*, Lane I: H7F-3A + H7F-3B(-10)*. Biotinylated H7F-3 and H7F-3A were used for ELONA. HS, human serum.


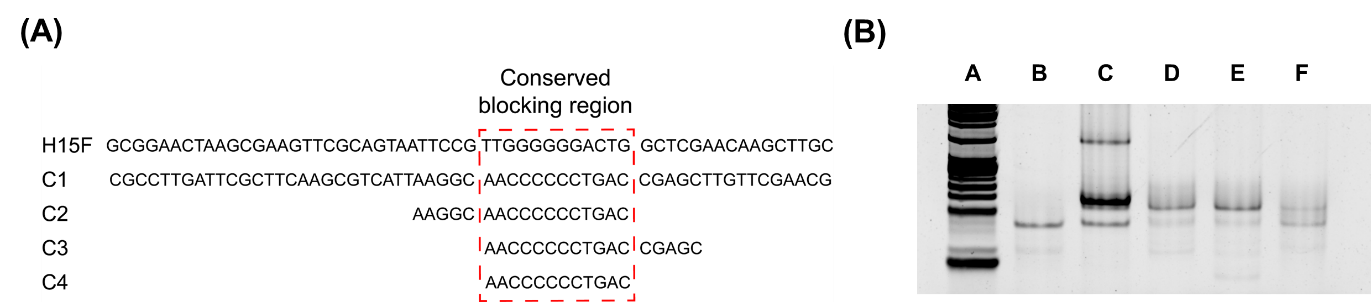


**Figure S9. Feasibility of the blocking strategy using the H15F aptamer.** (A) Sequence alignment of the H15F aptamer and its blocker DNAs (C1, C2, C3, and C4). The red box indicates a conserved blocking region. (B) Native PAGE analysis to confirm the hybridization of H15F aptamer and its blocker DNAs without target HT29 sEVs. Lane A: DNA ladder, Lane B: H15F, Lane C: H15F + C1, Lane D: H15F + C2, Lane E: H15F + C3, Lane F: H15F + C4. PAGE, polyacrylamide gel electrophoresis; sEV, small extracellular vesicle.


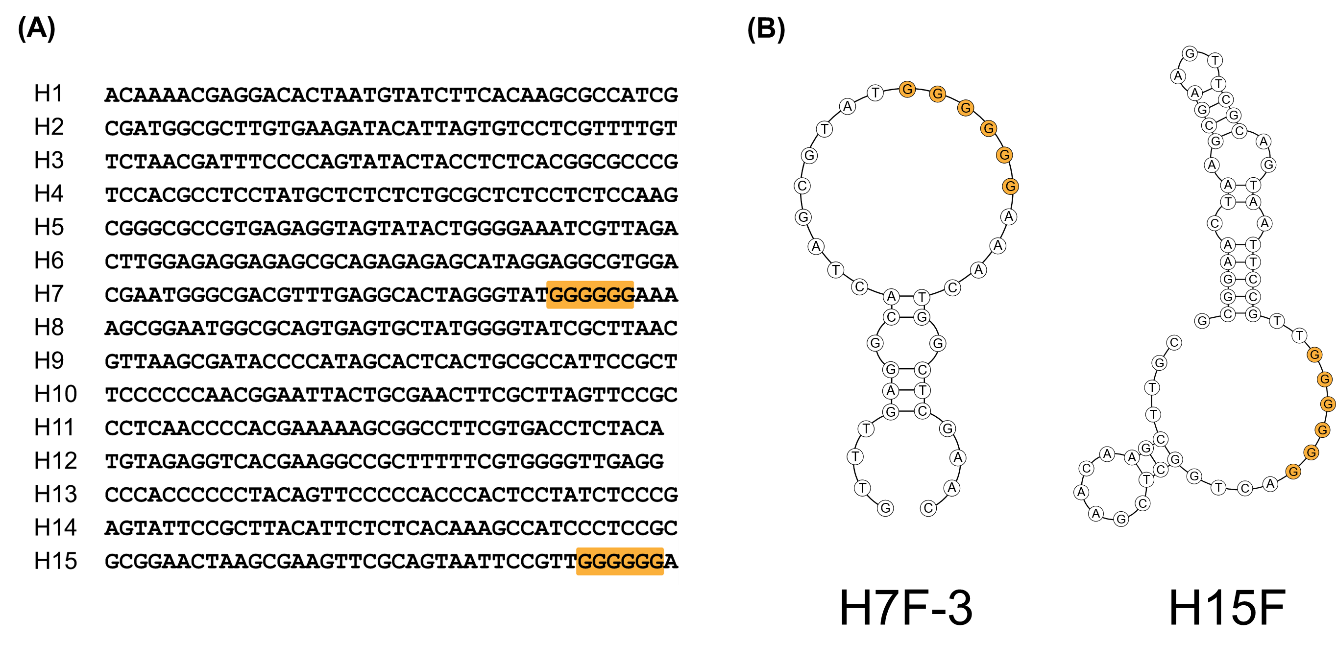


**Figure S10. Sequence analysis of the G6 motif.** (A) Sequence alignment of the top 15 aptamer candidates. (B) Conserved G6 motif sequence of the H7F-3 and H15F aptamers. The yellow highlight indicates the G6 motif sequence.


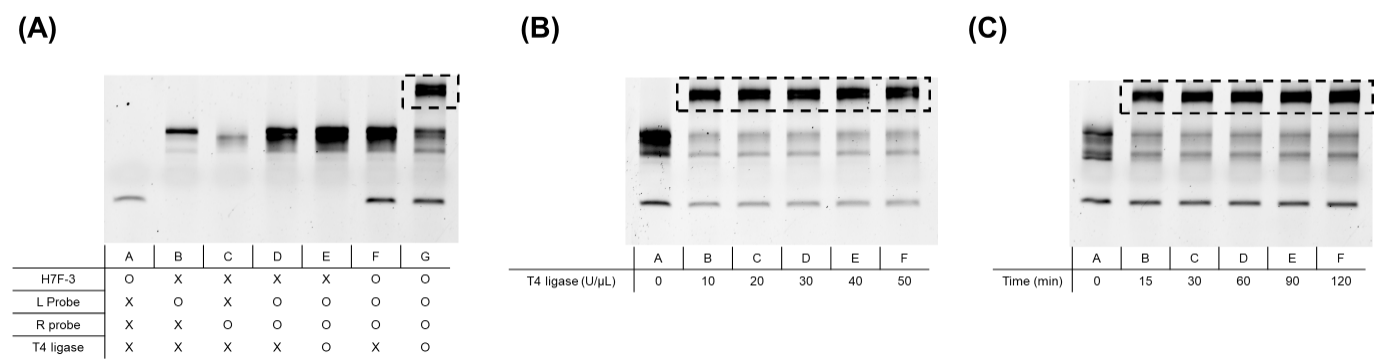


**Figure S11.** **T4 ligation feasibility and optimization of the ABLE system via denaturing PAGE analysis.** (A) Validation of T4 ligation. (B) Optimization of T4 ligase concentration. (C) Optimization of T4 ligation time. The black dashed boxes indicate the ligation products. ABLE, aptamer-based loop-mediated isothermal amplification for sEV detection; PAGE, polyacrylamide gel electrophoresis; sEV, small extracellular vesicle.


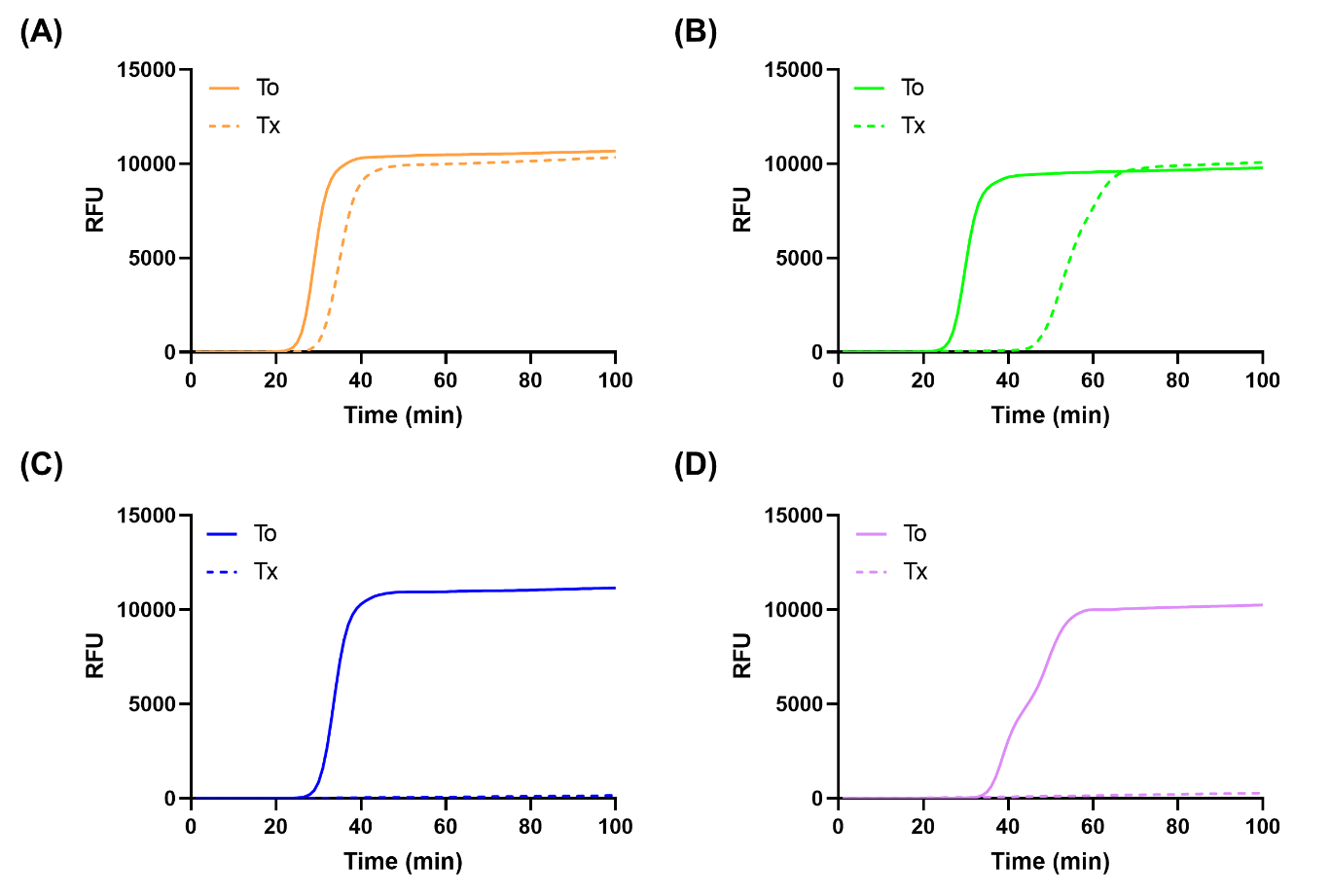


**Figure S12. Hairpin probe concentration optimization of the ABLE system.** Various concentrations (A: 10 nM, B: 1 nM, C: 100 pM, and D: 10 pM) of hairpin probes (L-loop and R-loop) were used for optimization. “To” represents samples with the H7F-3 aptamer and “Tx” represents samples without the H7F-3 aptamer. ABLE, aptamer-based loop-mediated isothermal amplification for sEV detection; sEV, small extracellular vesicle.
